# Supplementary material for: Single-cell multiomics reveals the interplay of clonal evolution and cellular plasticity in hepatoblastoma
Source: Nat Commun. 2024 Apr 8;15:3031. doi: 10.1038/s41467-024-47280-x (PMC11001886; doi:10.1038/s41467-024-47280-x)
Supplement: Supplementary file 1 — Supplementary Information [file 41467_2024_47280_MOESM1_ESM.pdf]

Supplementary Figure 1

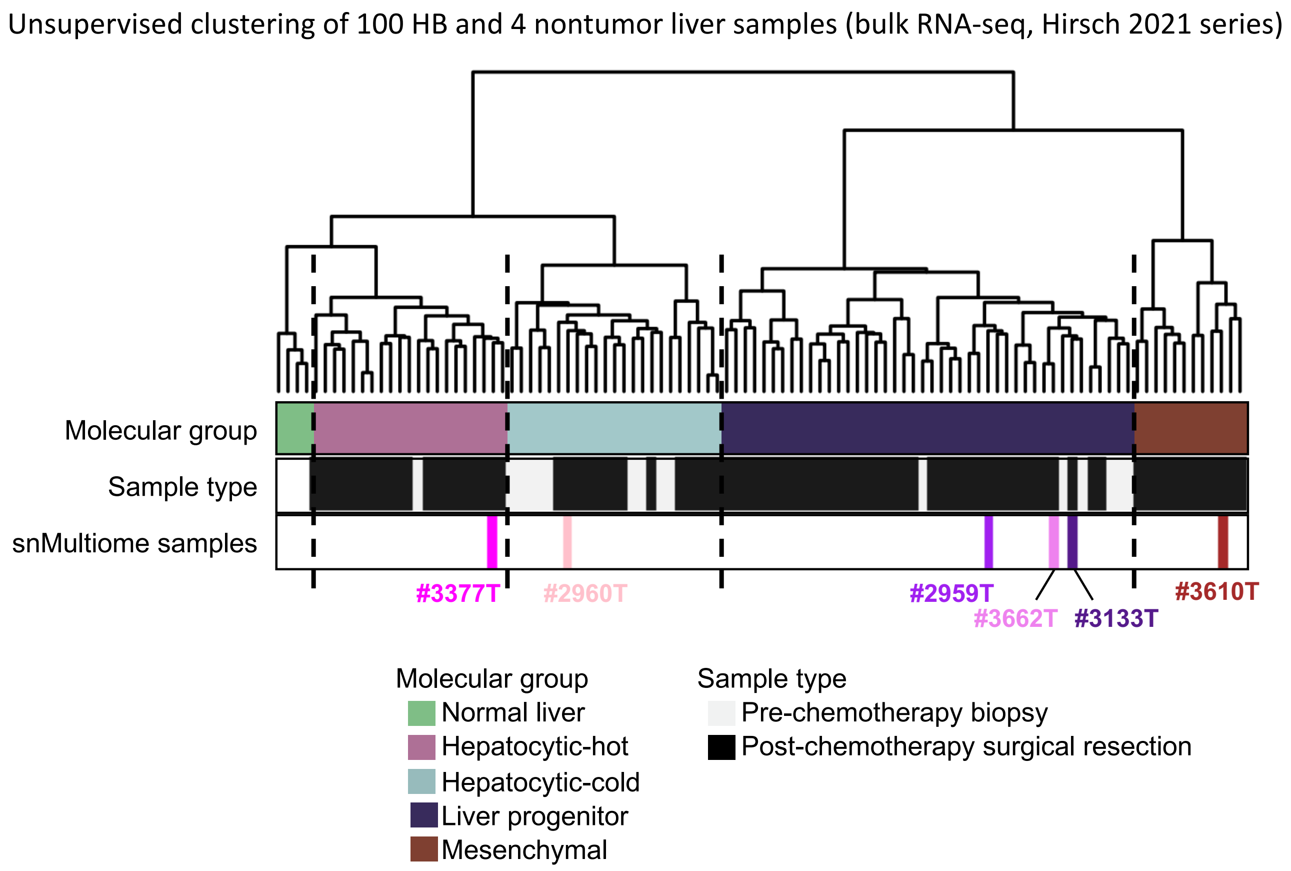

Supplementary Figure 1. Transcriptomic classification of the 6 samples selected for single-nucleus Multiome. The dendogram represents the hierarchical clustering of bulk RNA-seq profiles of 100 HB and 4 non-tumor liver samples (Hirsch data set). The 6 samples selected for snMultiome are indicated below, together with bulk molecular groups from the original study and sample type (pre / post-chemotherapy).

Supplementary Figure 2

#2959T

FFPE section / miror block / 100% tumoral
70% Embryonal component intricated with 30% Fetal component with some intermediate pattern / rosettes / No hematopoiesis / No infllammation.

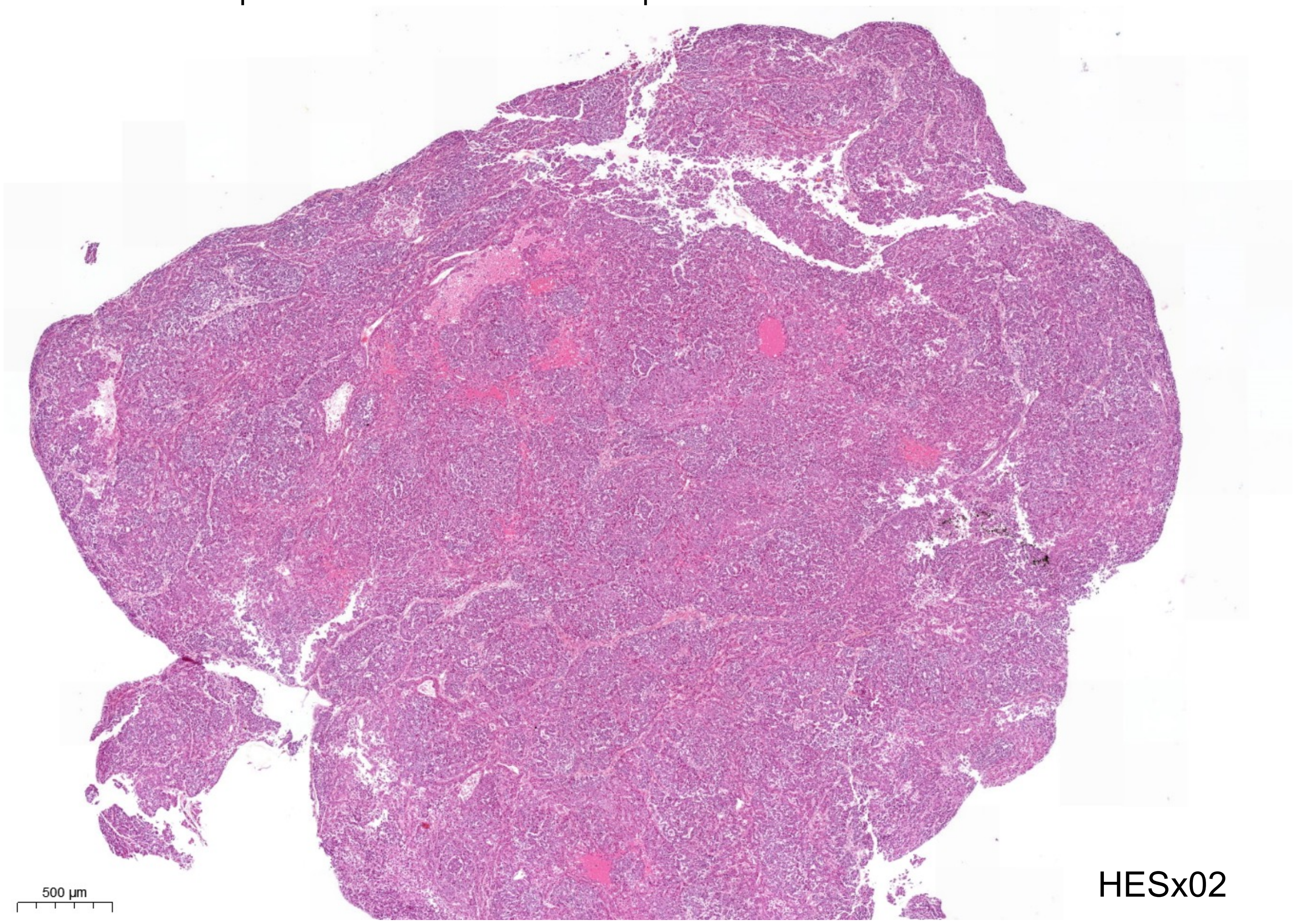

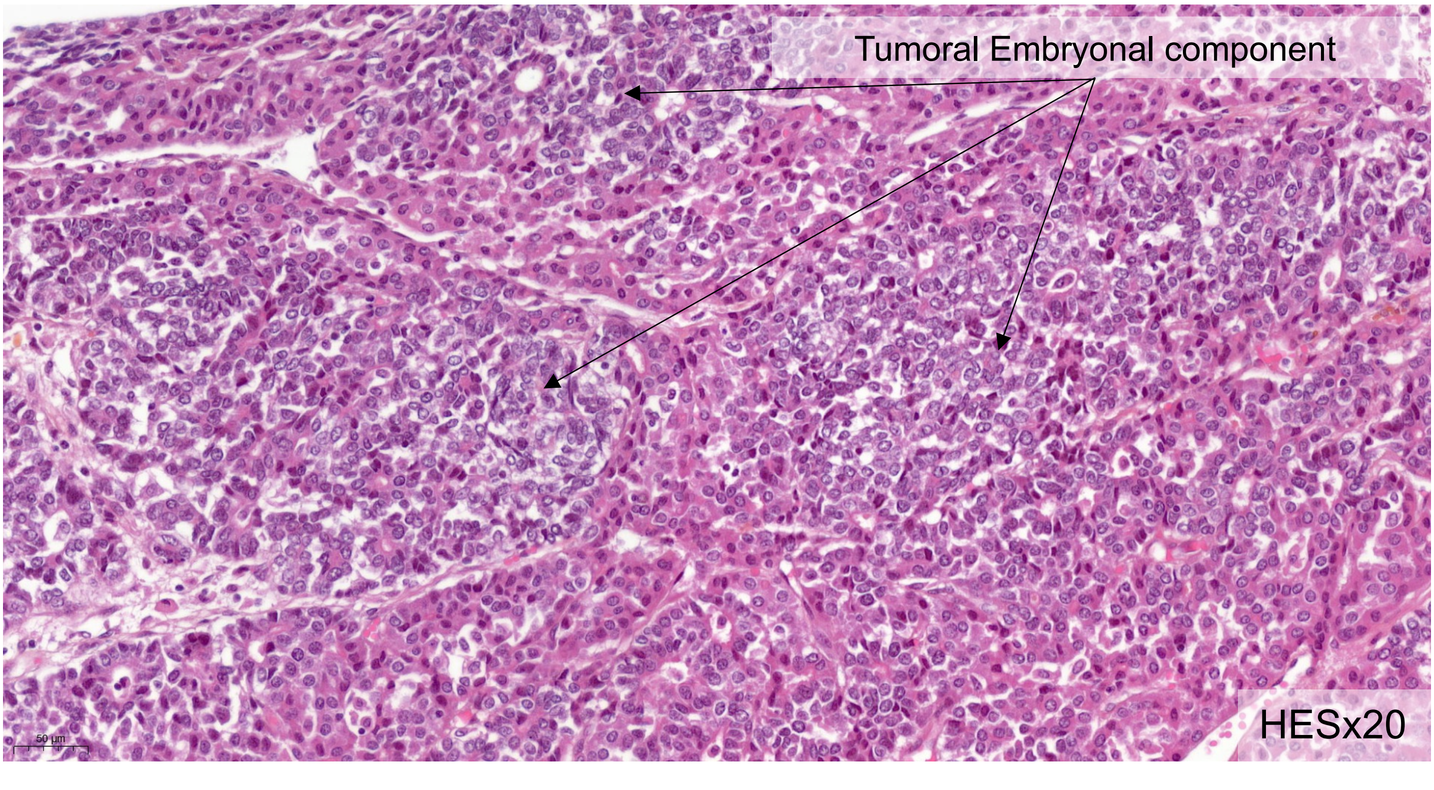

Supplementary Figure 2. Mirror block reviewing of the 6 cases analyzed by single-nucleus Multiome.

Supplementary Figure 2 (continued)

#2960T

FFPE section / miror block /
100% Tumoral Fetal component / pseudoacinous pattern + few bile plugs /
Lymphocyte inflitrate ++ / small arterioles within fibrotic spots / No hematopoiesis.

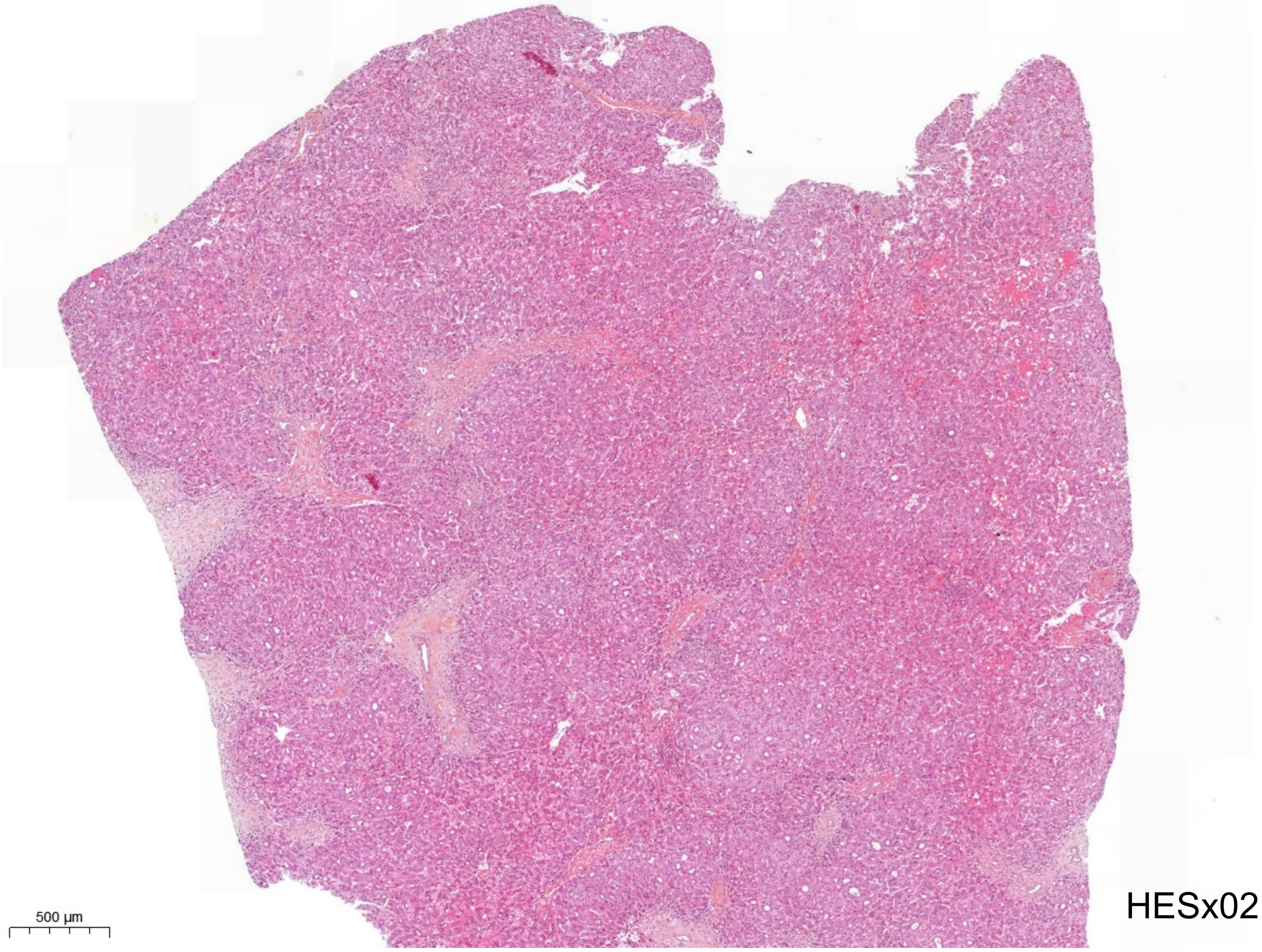

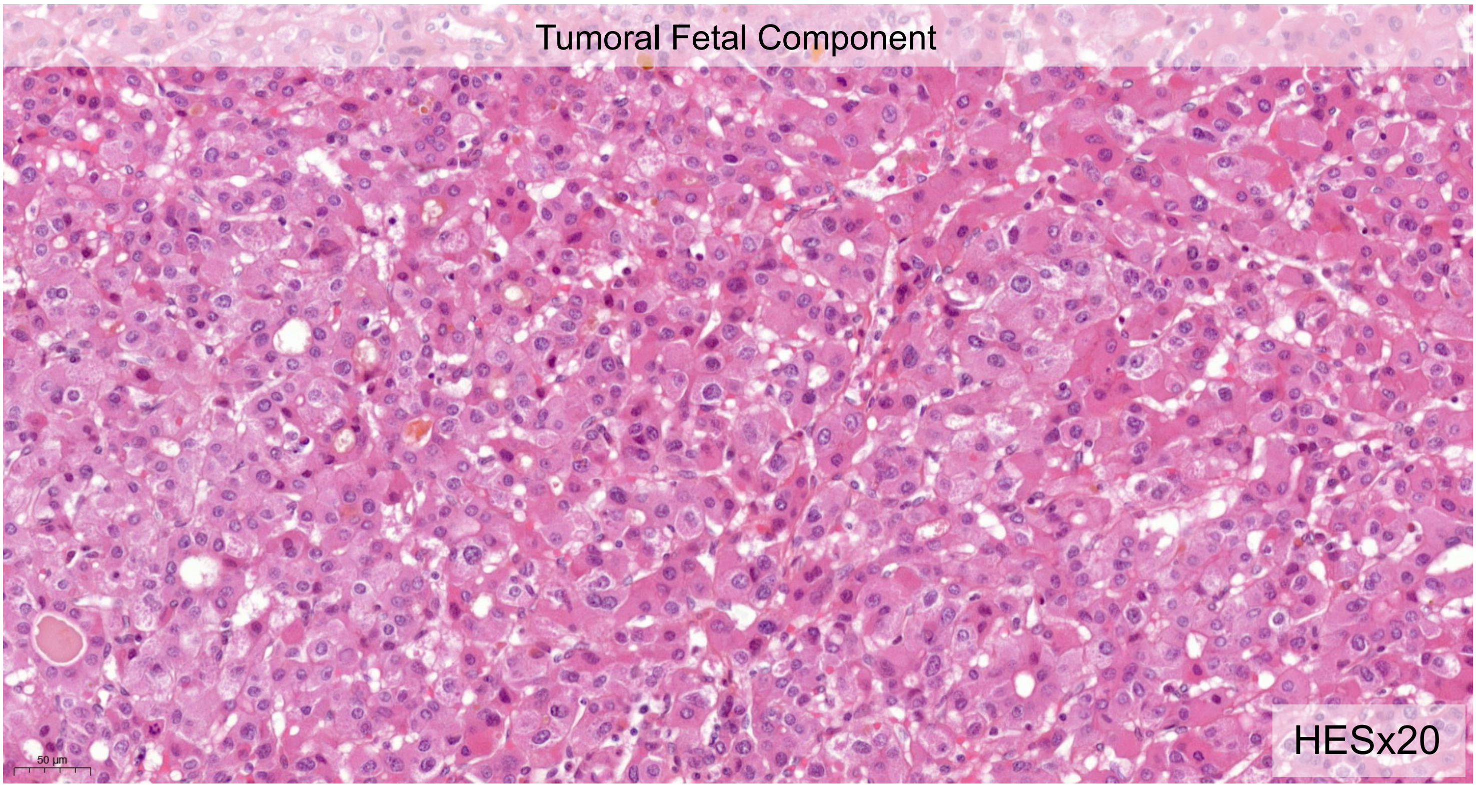

Supplementary Figure 2. Mirror block reviewing of the 6 cases analyzed by single-nucleus Multiome.

Supplementary Figure 2 (continued)

#3133T

FFPE section / miror block /
70% Embryonal intricated with 30% Fetal with some intermediate pattern / rosettes /
No hematopoiesis / No inflammation / 5% fibrosis.

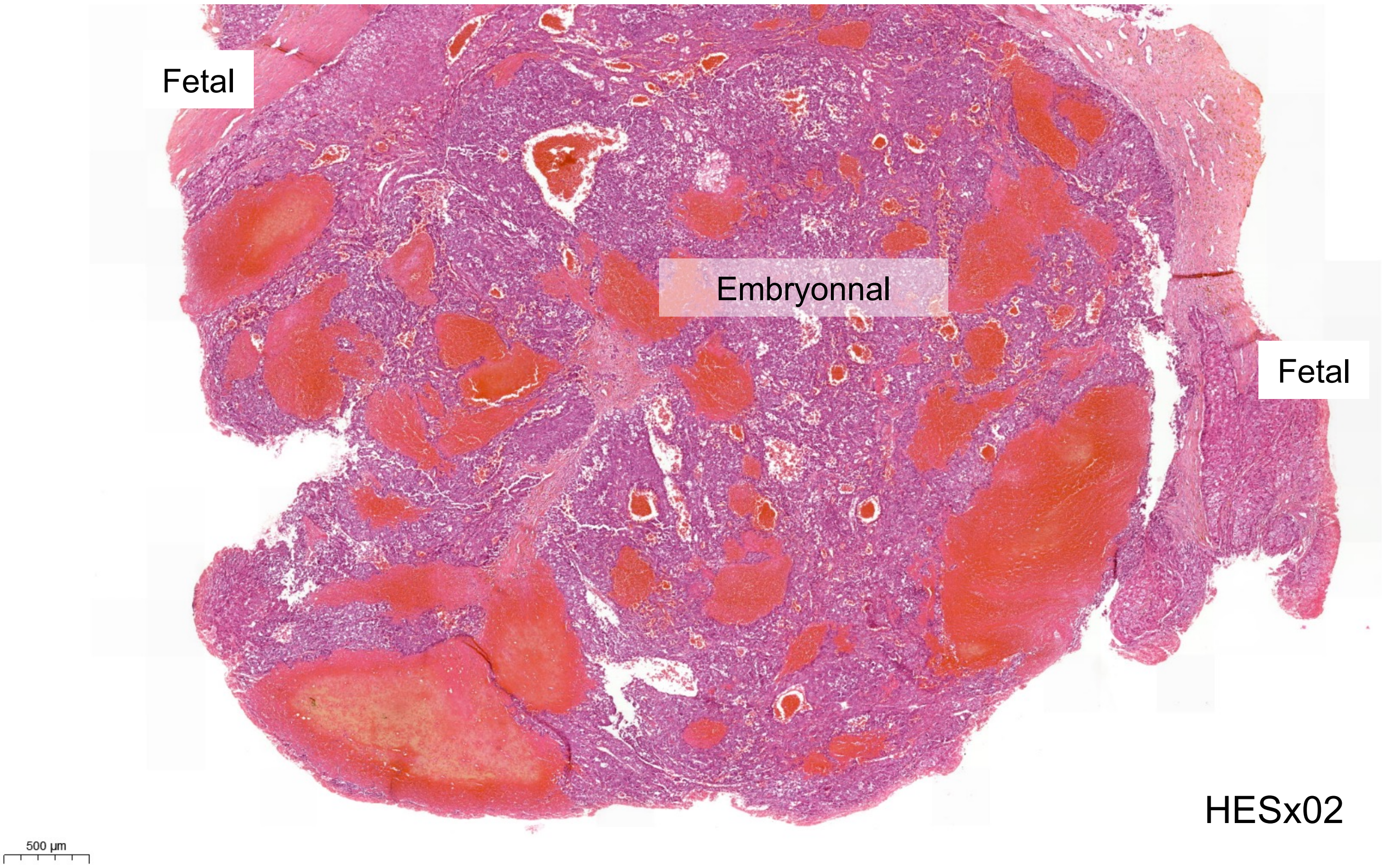

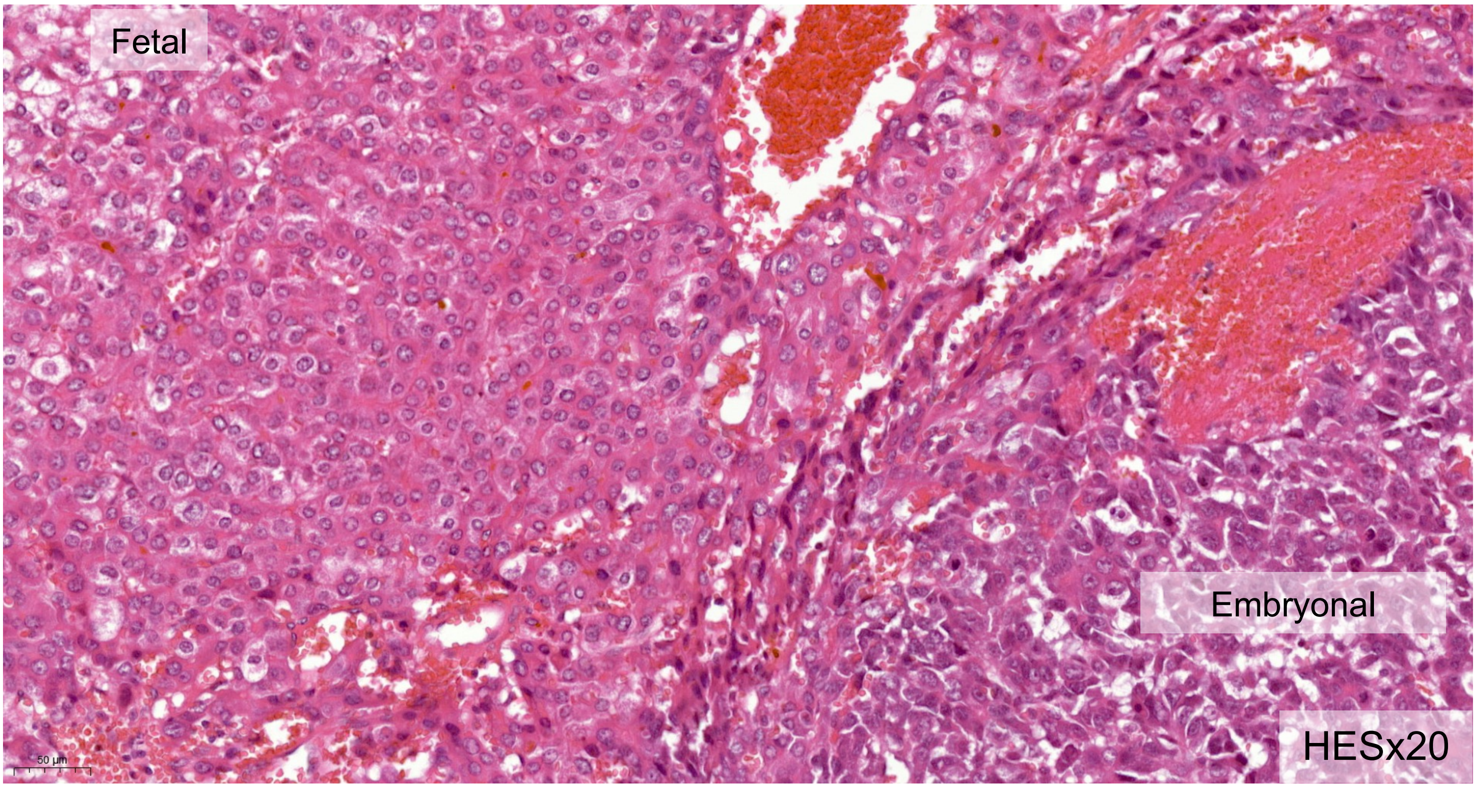

Supplementary Figure 2. Mirror block reviewing of the 6 cases analyzed by single-nucleus Multiome.

Supplementary Figure 2 (continued)

#3377T

FFPE section / miror block /
100% Tumoral Fetal component

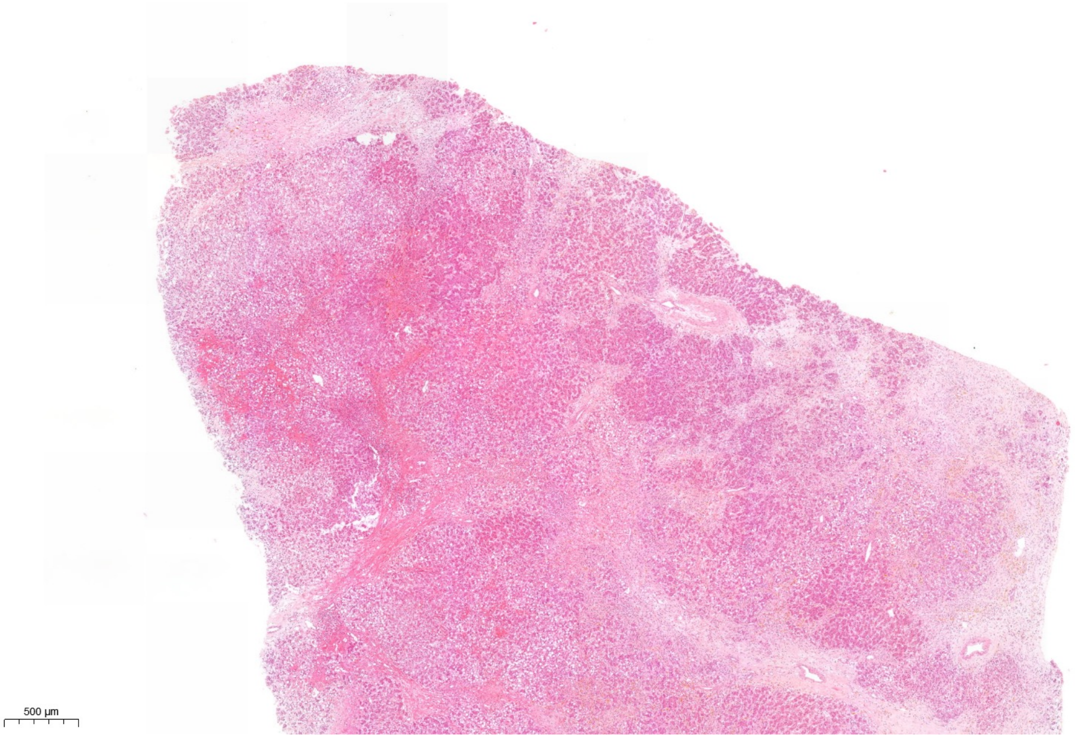

HESx02

100% Tumoral Fetal component
Steatosis / pseudoacinous pattern + few bile plugs + little trabecular trend spots /
Lymphocytes macrophages siderophages inflitrate + / 2 hematopoietic spots / few arterioles

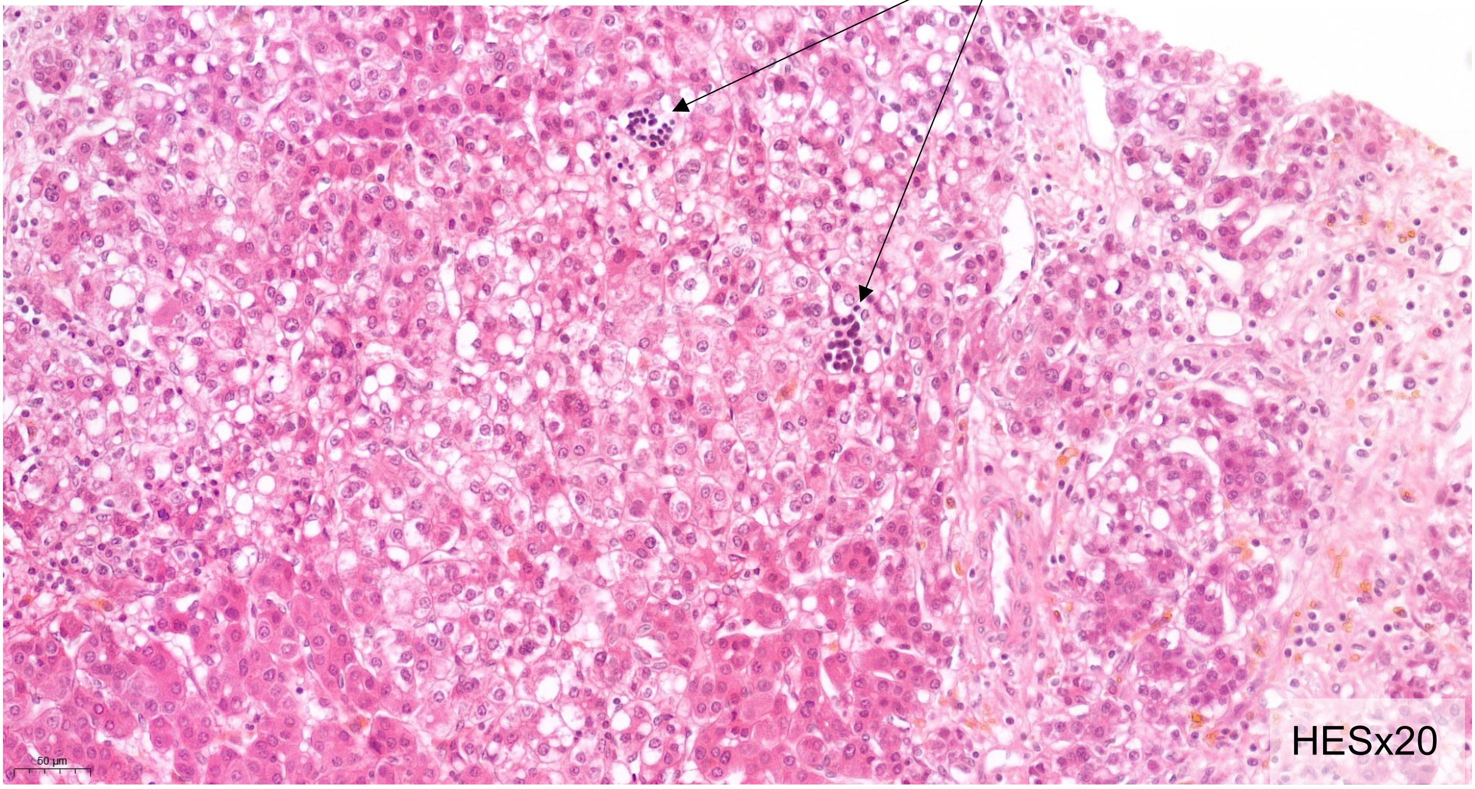

Supplementary Figure 2. Mirror block reviewing of the 6 cases analyzed by single-nucleus Multiome.

Supplementary Figure 2 (continued)

#3610T

Frozen section / Hamamatsu C13210 digitalization x40

100% Tumoral mesenchymal component (80% immature mesenchyma + 20% osteoid)

No hematopoiesis / No or scarce inflammation.

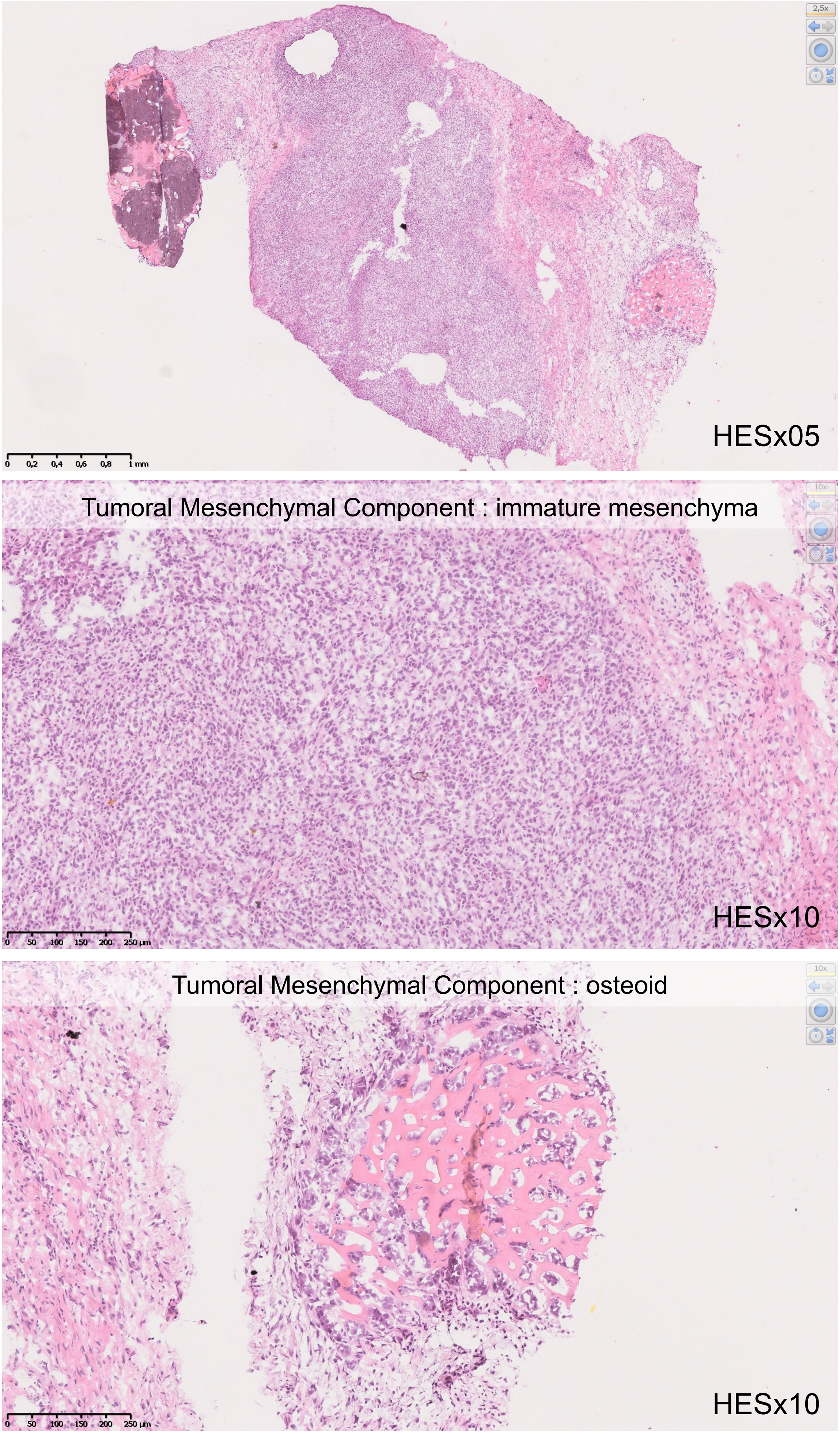

Supplementary Figure 2. Mirror block reviewing of the 6 cases analyzed by single-nucleus Multiome.

Supplementary Figure 2 (continued)

#3662T

FFPE section / miror block /
60 % tumor / 40% fibrosis

Fetal with some vascular bloody spaces, few pseudoacinous pattern + rare bile plugs / Embryonal with rares rosettes intricated with a little Fetal with some intermediate pattern +/- trabecular / thrombosed vessels, siderophages++ lymphocytes+ few calcifications / No obvious hematopoiesis.

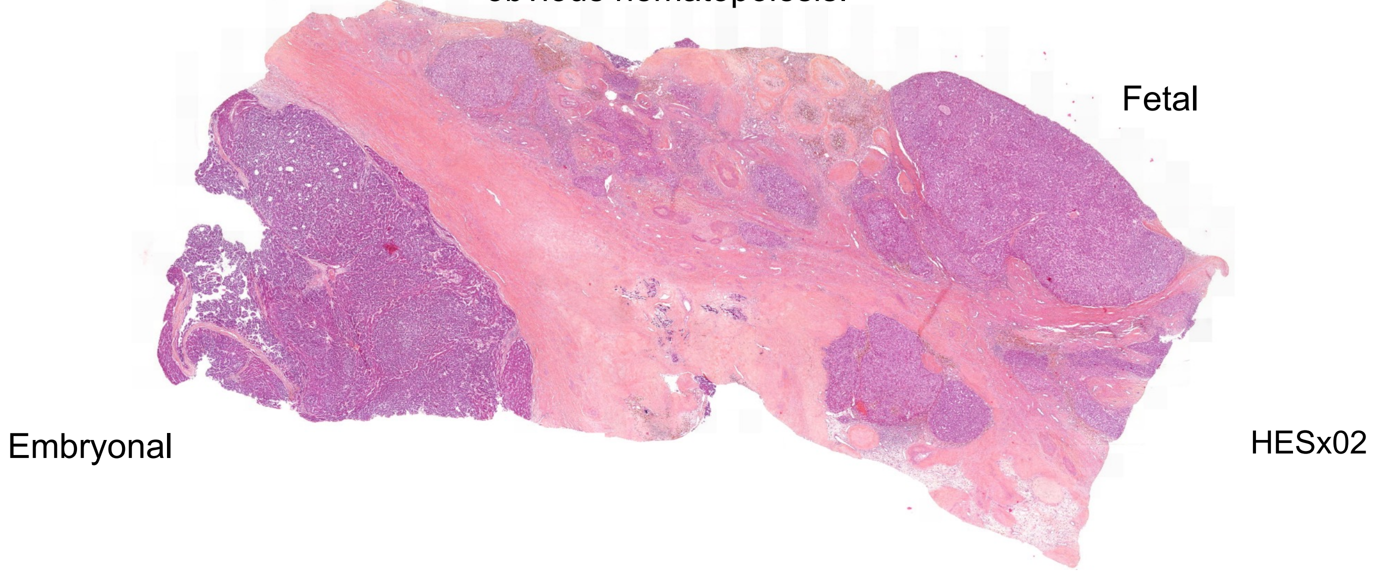

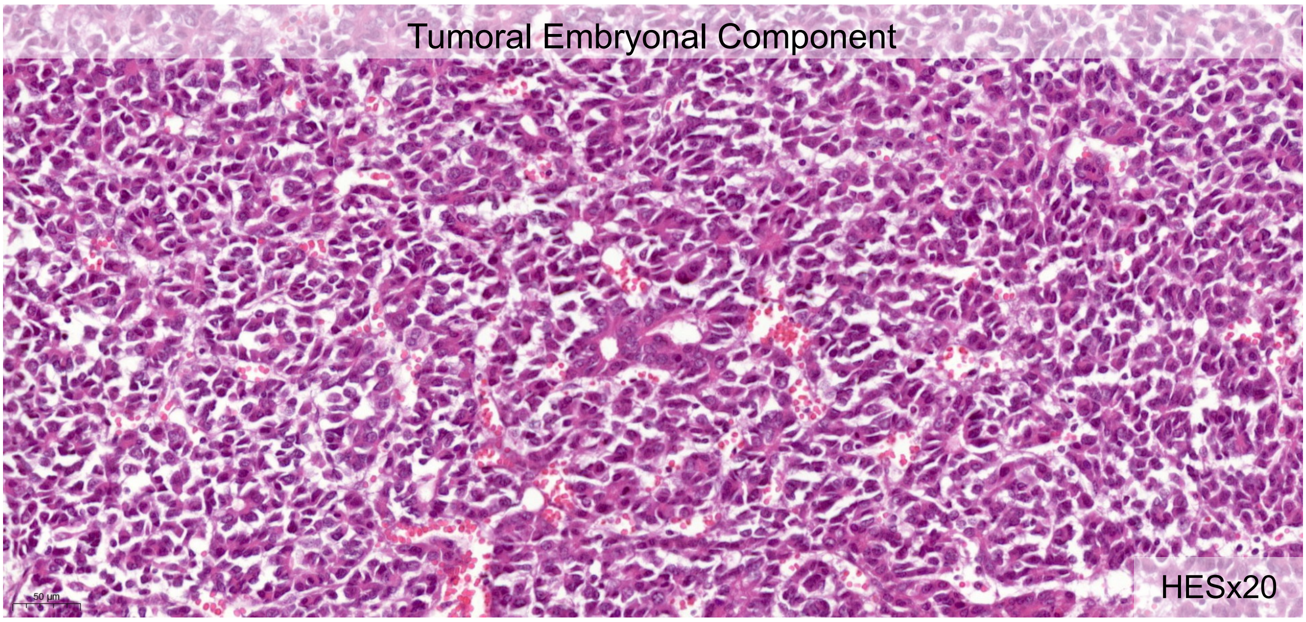

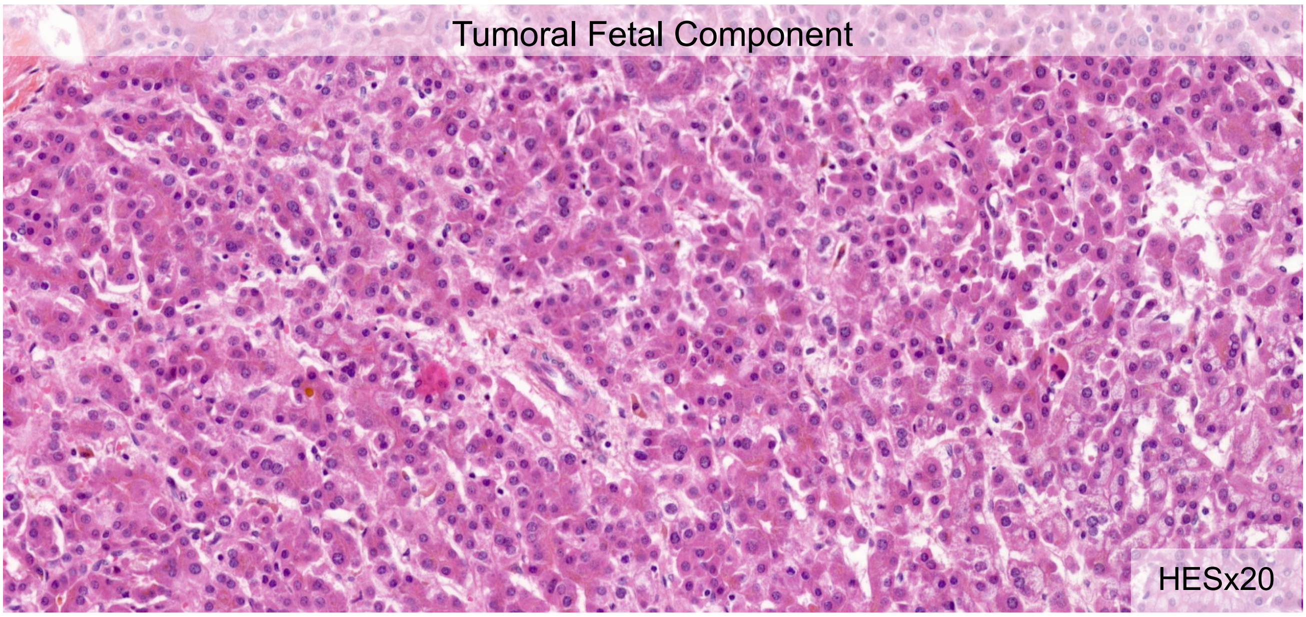

Supplementary Figure 2. Mirror block reviewing of the 6 cases analyzed by single-nucleus Multiome.

Supplementary Figure 3

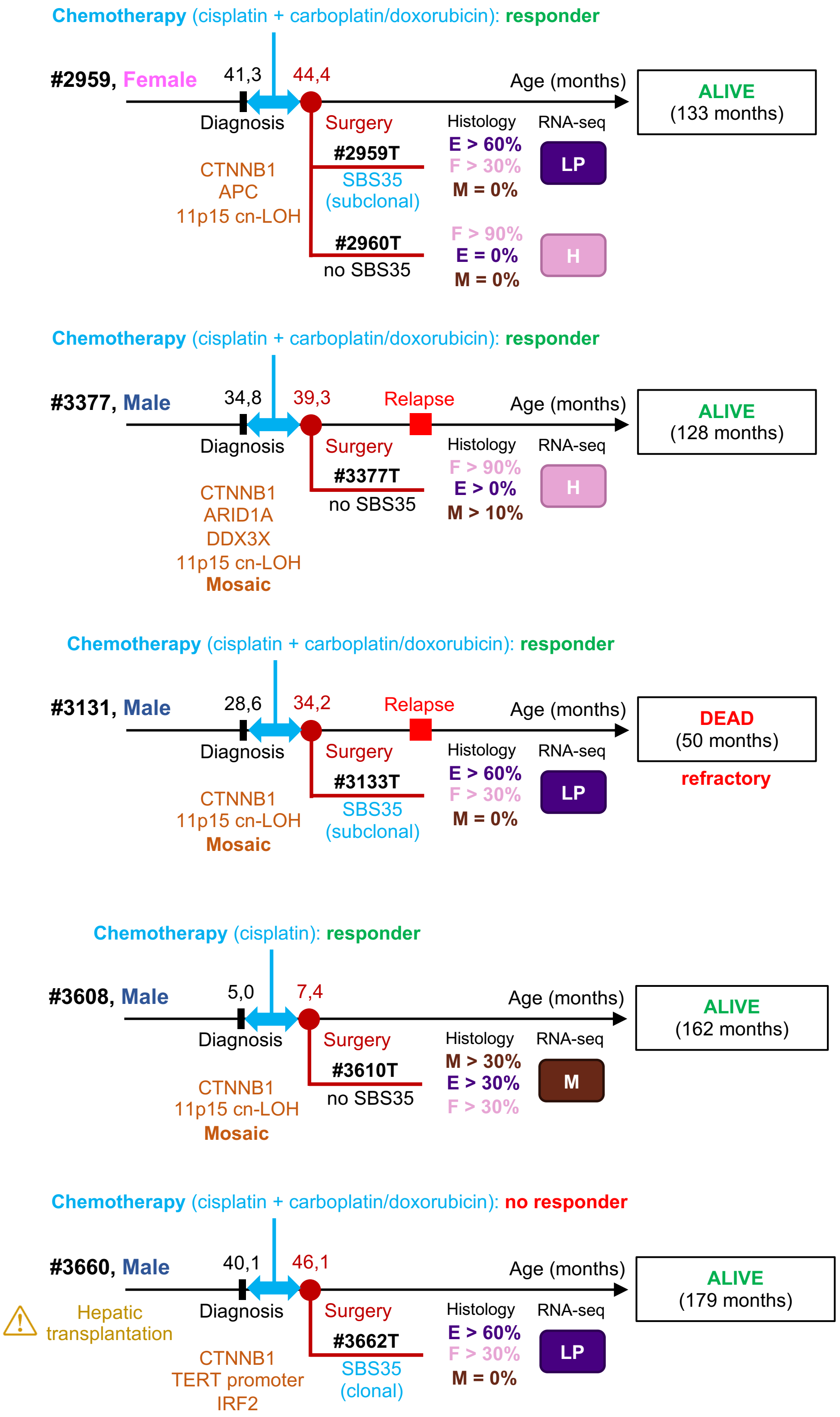

Supplementary Figure 3. Clinical and molecular characteristics of the 6 tumors analyzed by WGS and scMultiome. The clinical history of each patient is represented, with age at diagnosis, surgery and last news status (in months). The histological composition of each tumor is indicated (E: embryonal, F: fetal, M: mesenchymal). Molecular annotations include genetic drivers identified by WGS, transcriptomic subgroup identified by bulk RNA-seq (H: Hepatocytic, LP: Liver Progenitor, M: Mesenchymal), and the presence of the cisplatin-induced mutational signature SBS35.

Supplementary Figure 4

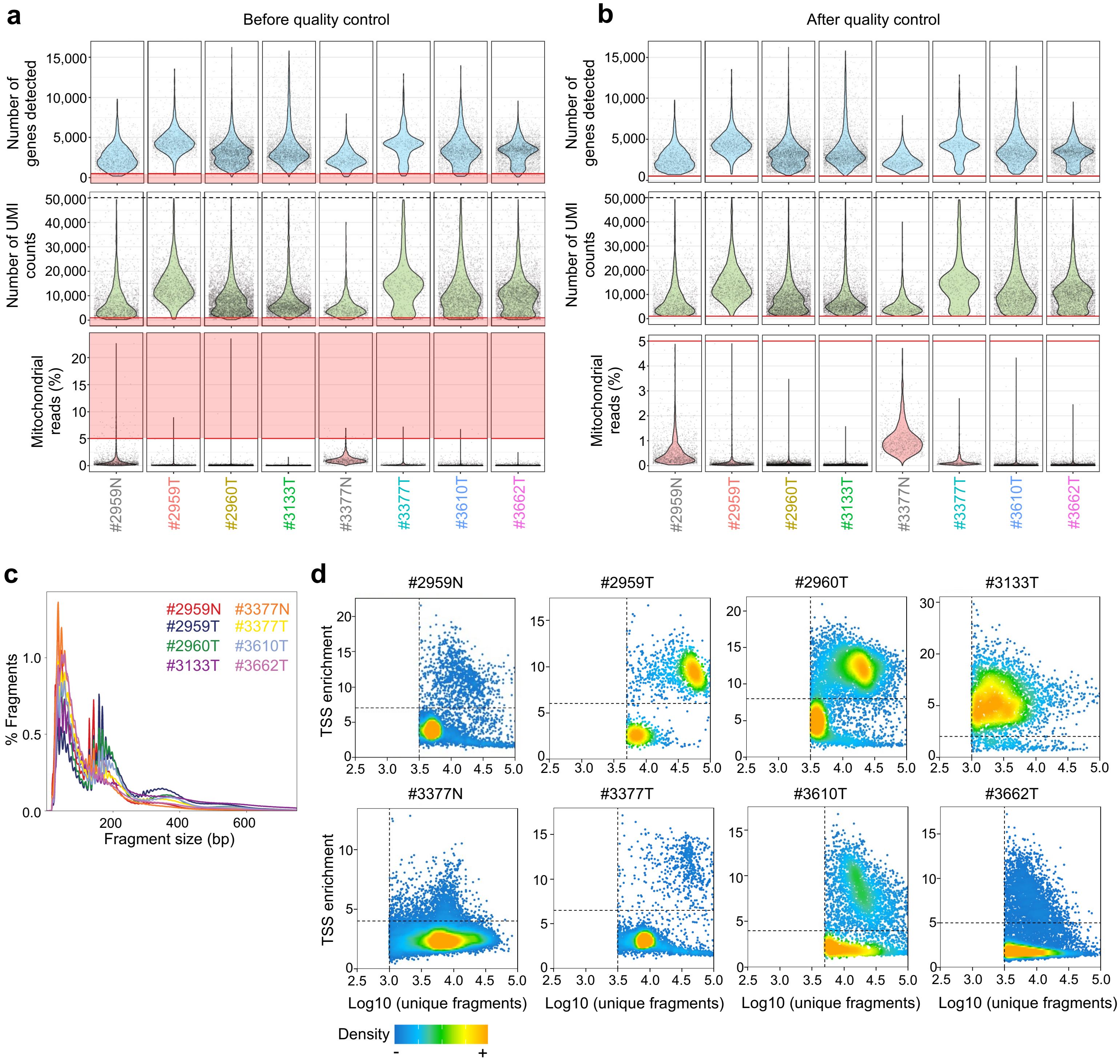

Supplementary Figure 4. Quality metrics of the single-cell Multiome dataset. a snRNA-seq metrics before quality control: number of genes detected per cell (top), number of UMI counts per cell (middle) and proportion of mitochondrial reads (bottom). Red lines indicate the thresholds used for cell filtering. Dotted lines indicate that the graphs were cut. **b** Same as **a** after quality control. **c** Distribution of snATAC-seq fragment size for all 8 samples (each color represents a sample). **d** Number of unique snATAC-seq fragments and transcription start site (TSS) enrichment of snATAC-seq reads for each sample. Dotted lines represent the thresholds used for cell filtering.

Supplementary Figure 5

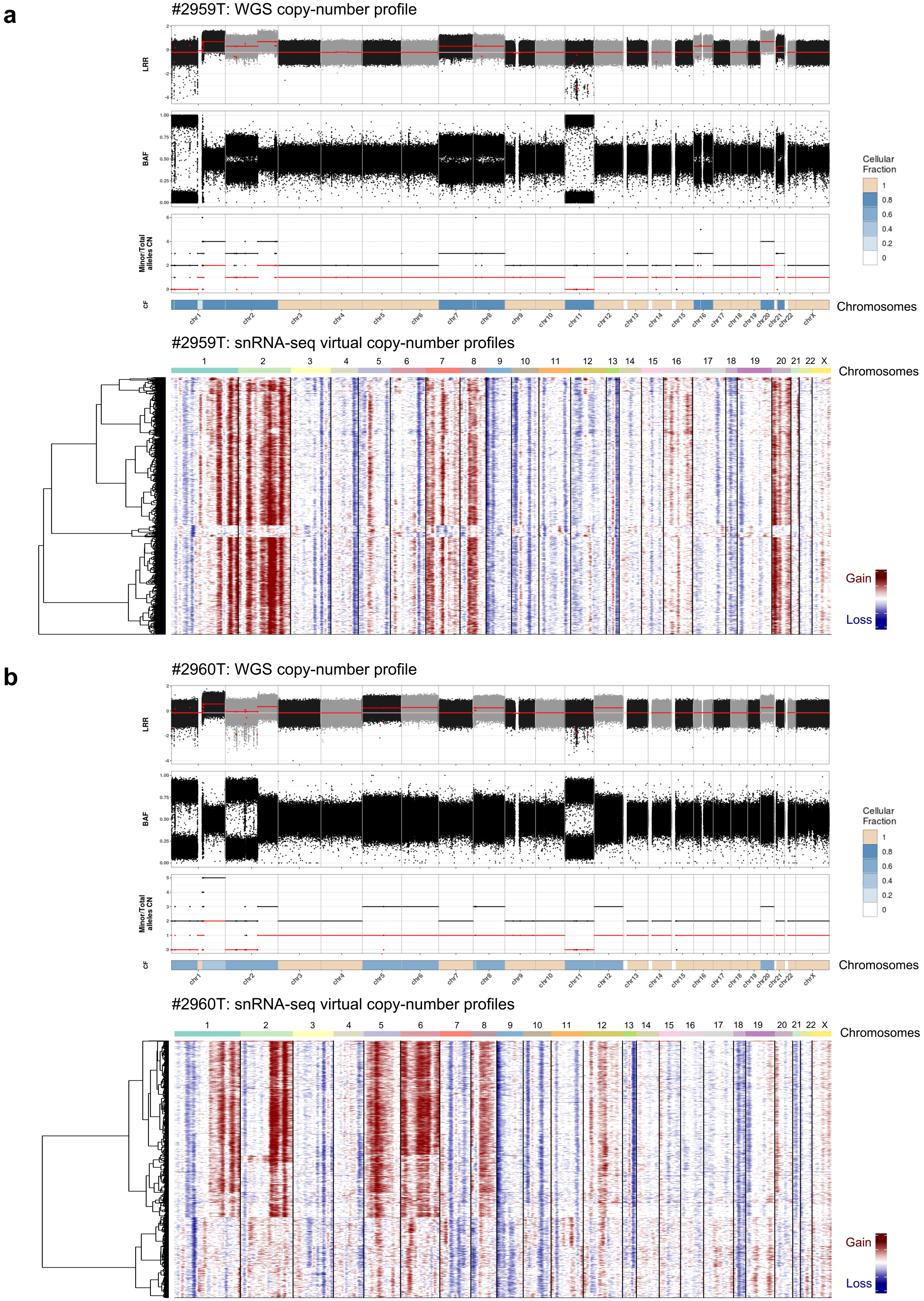

Supplementary Figure 5. Copy-number profiles reconstructed from WGS and single-nucleus RNA-seq data. a Tumor #2959T. (Top) CNA profile reconstructed from WGS using *FACETS*. Coverage log-ratio between the tumor and matched normal samples (LRR), B allele frequency (BAF) and absolute cop-number (minor: red, total: black) profiles are represented along the genome, together with the cellular fraction of each aberration. (Bottom) Virtual copy-number profiles reconstructed from snRNA-seq data using *InferCNV*. **b,c,d,e,f** Same as **a** for tumors #2960T, #3133T, #3377T, #3610T and #3662T.

Supplementary Figure 5 (continued)

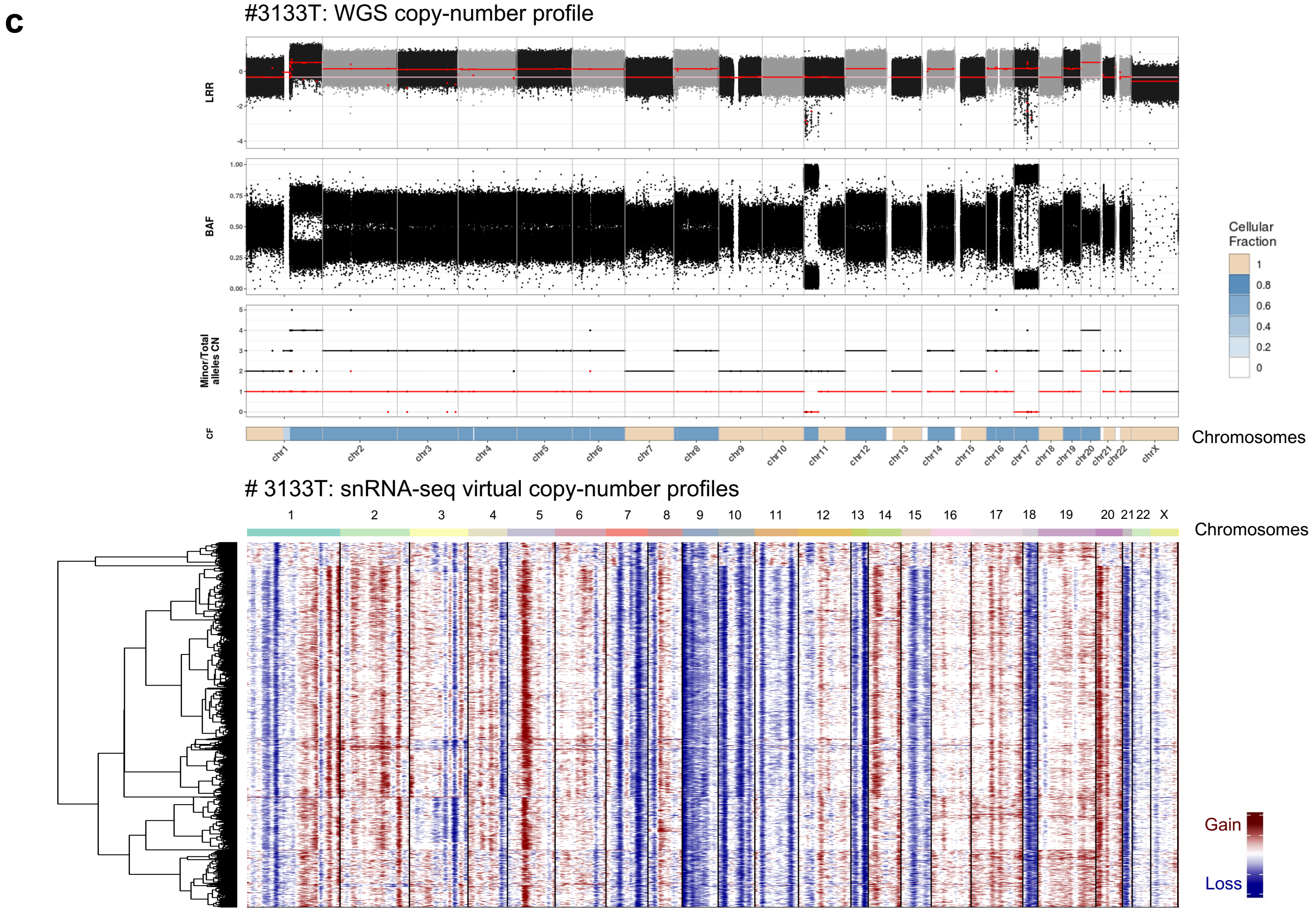

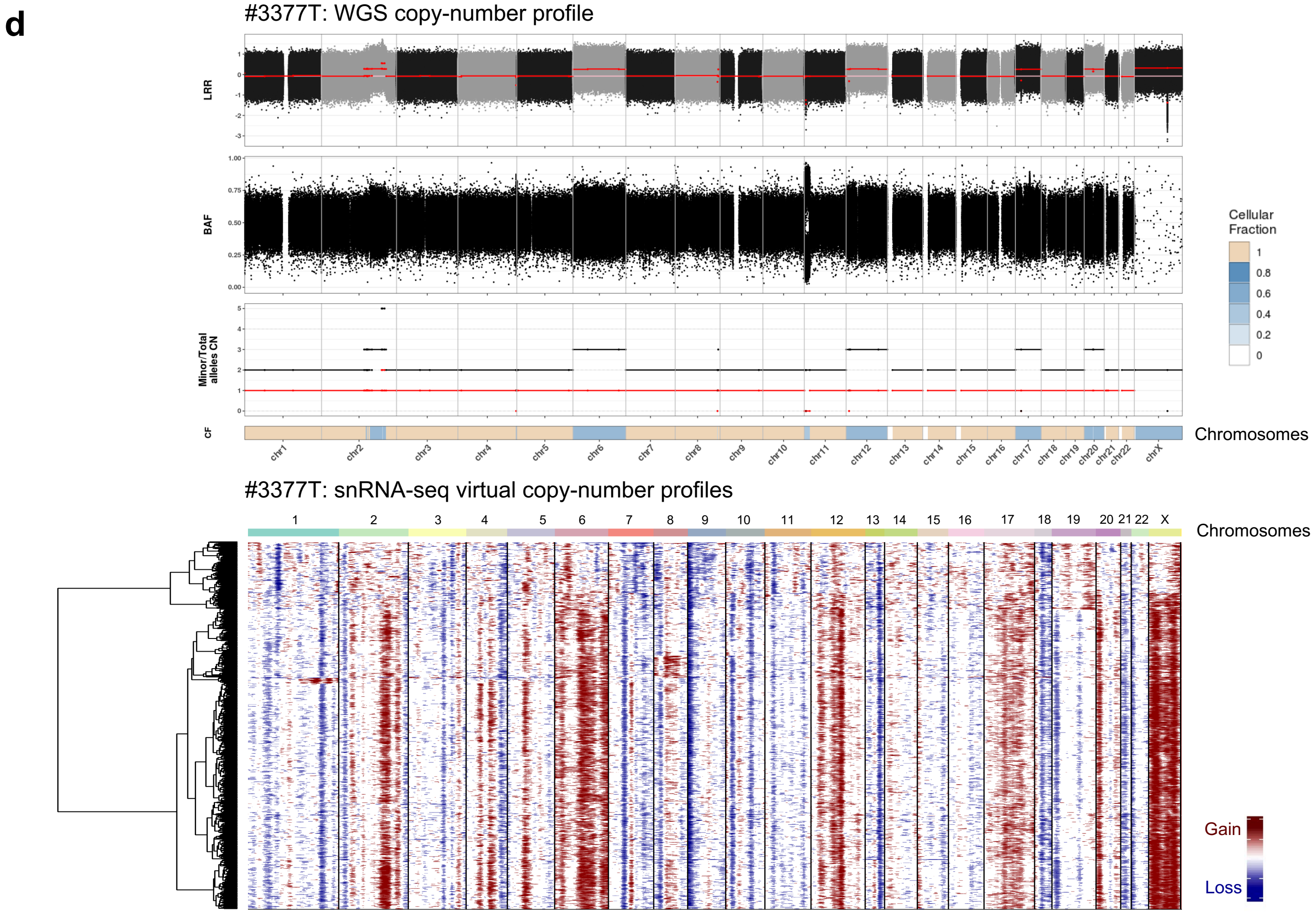

Supplementary Figure 5. Copy-number profiles reconstructed from WGS and single-nucleus RNA-seq data. a Tumor #2959T. (Top) CNA profile reconstructed from WGS using *FACETS*. Coverage log-ratio between the tumor and matched normal samples (LRR), B allele frequency (BAF) and absolute cop-number (minor: red, total: black) profiles are represented along the genome, together with the cellular fraction of each aberration. (Bottom) Virtual copy-number profiles reconstructed from snRNA-seq data using *InferCNV*. **b,c,d,e,f** Same as **a** for tumors #2960T, #3133T, #3377T, #3610T and #3662T.

Supplementary Figure 5 (continued)

e

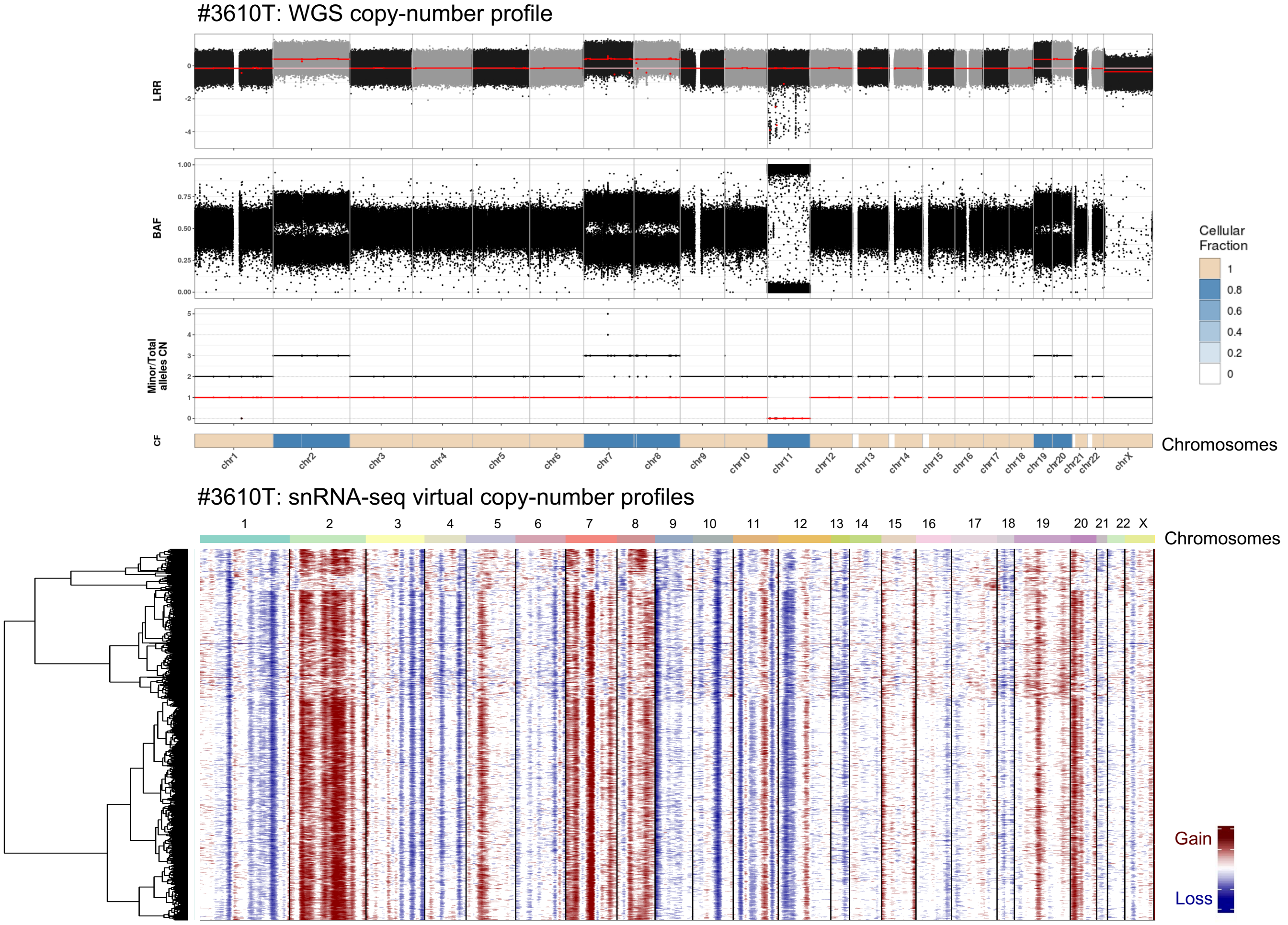

f

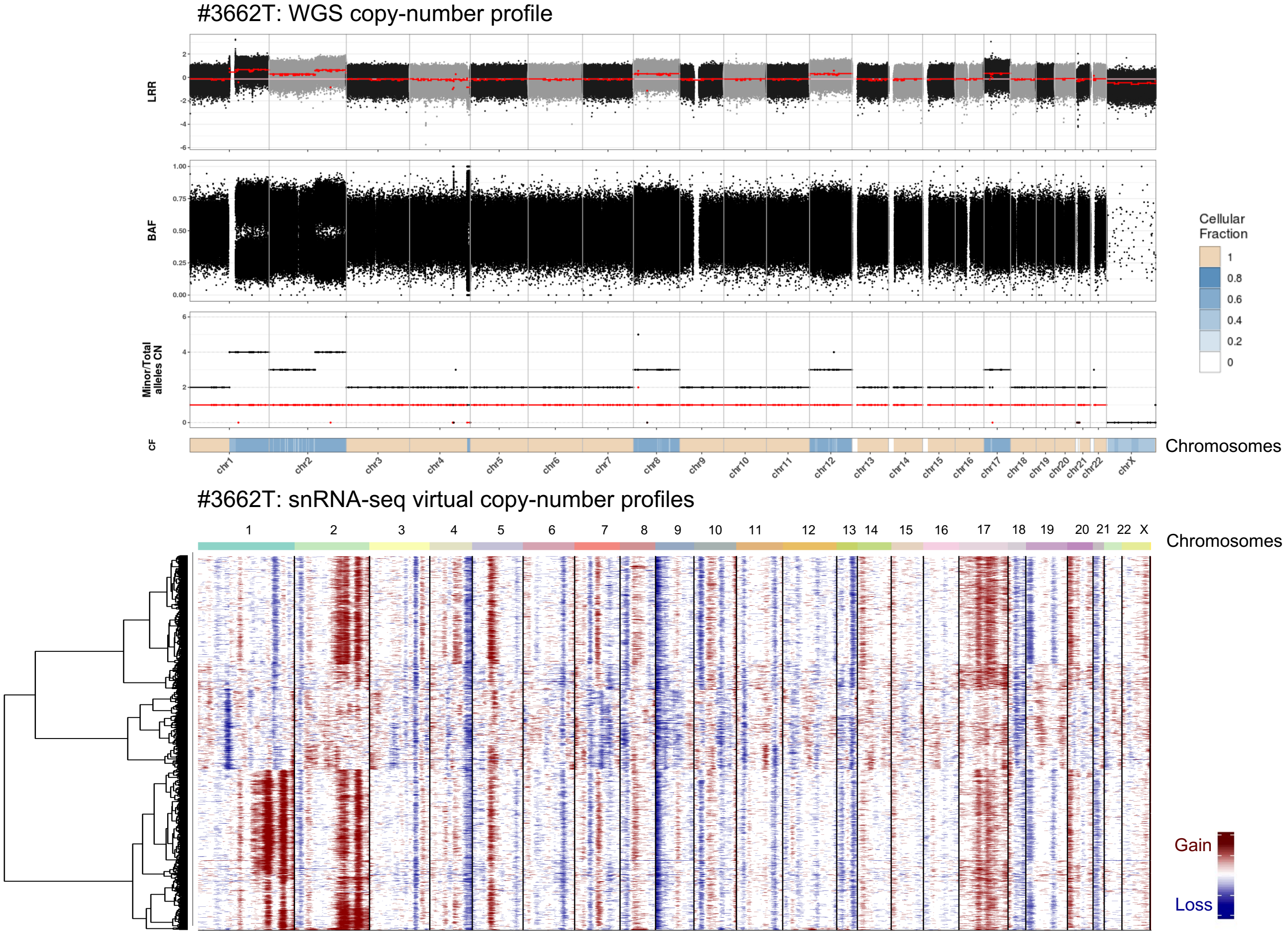

Supplementary Figure 5. Copy-number profiles reconstructed from WGS and single-nucleus RNA-seq data. a Tumor #2959T. (Top) CNA profile reconstructed from WGS using *FACETS*. Coverage log-ratio between the tumor and matched normal samples (LRR), B allele frequency (BAF) and absolute cop-number (minor: red, total: black) profiles are represented along the genome, together with the cellular fraction of each aberration. (Bottom) Virtual copy-number profiles reconstructed from snRNA-seq data using *InferCNV*. **b,c,d,e,f** Same as **a** for tumors #2960T, #3133T, #3377T, #3610T and #3662T.

Supplementary Figure 5 (continued)

g

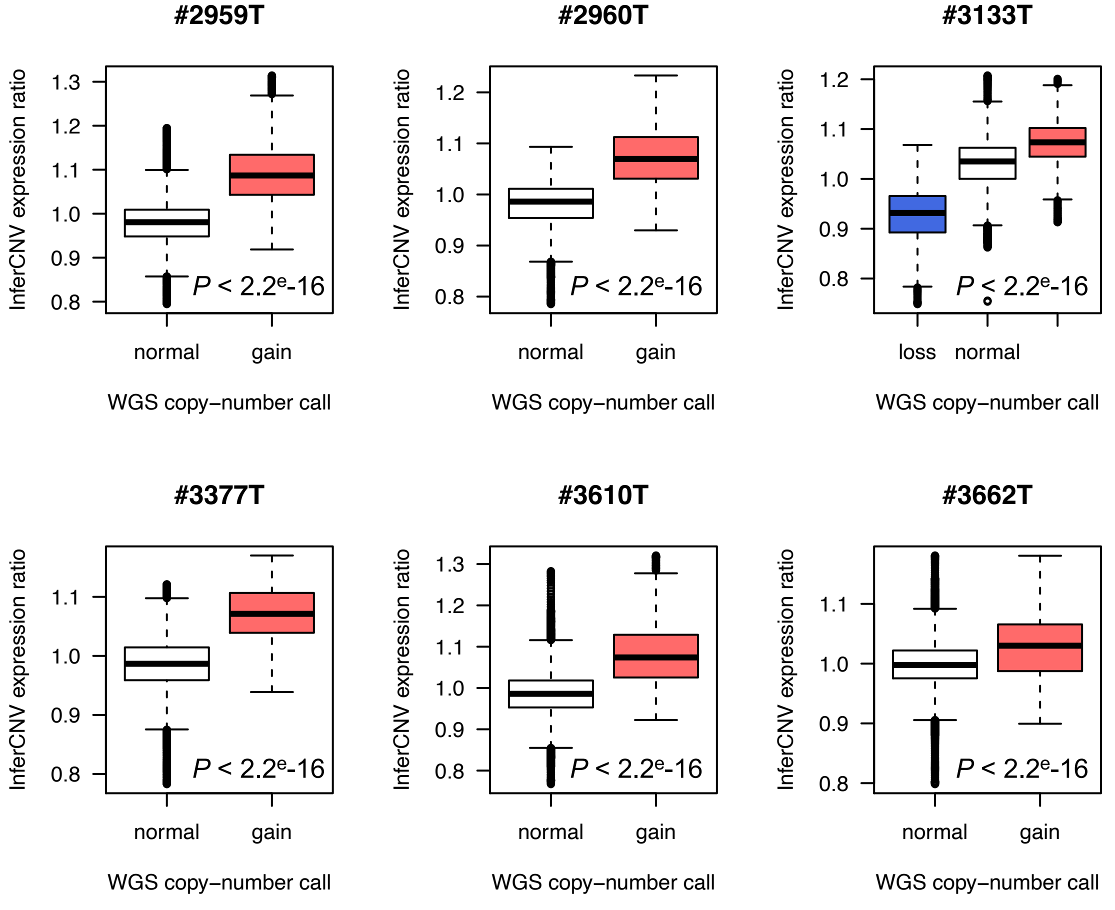

h

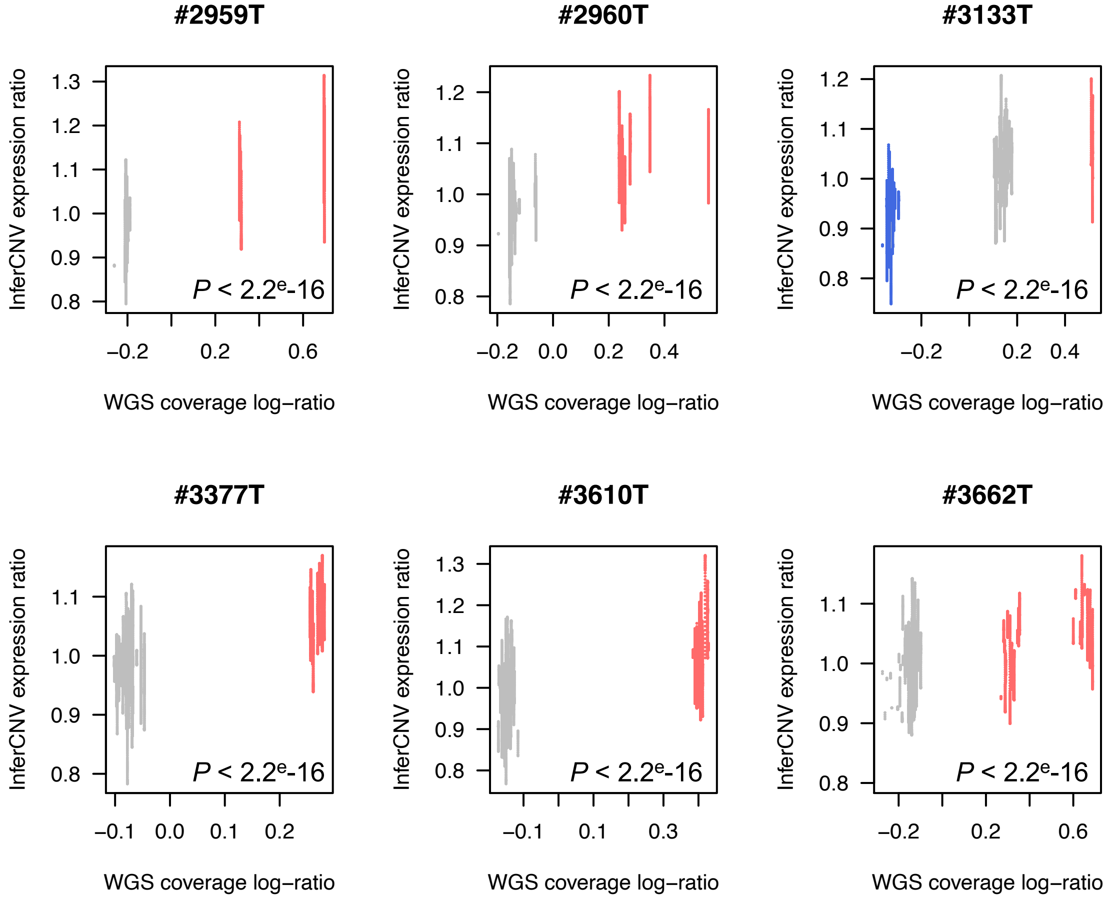

Supplementary Figure 5. Copy-number profiles reconstructed from WGS and single-nucleus RNA-seq data. g, Boxplots showing the distribution of InferCNV expression ratios as a function of the segment's copy-number status derived from WGS data. Middle bar, median; box, interquartile range; bars extend to 1.5 times the interquartile range. P-values were obtained using Wilcoxon rank sum tests (two-sided). Note that deletions were only detected in sample #3133T. **h,** Correlation between InferCNV expression ratios and WGS coverage log-ratio at the gene position. Source data are provided in the Source Data file. P-values were obtained using Pearson's correlation test (two-sided).

Supplementary Figure 6

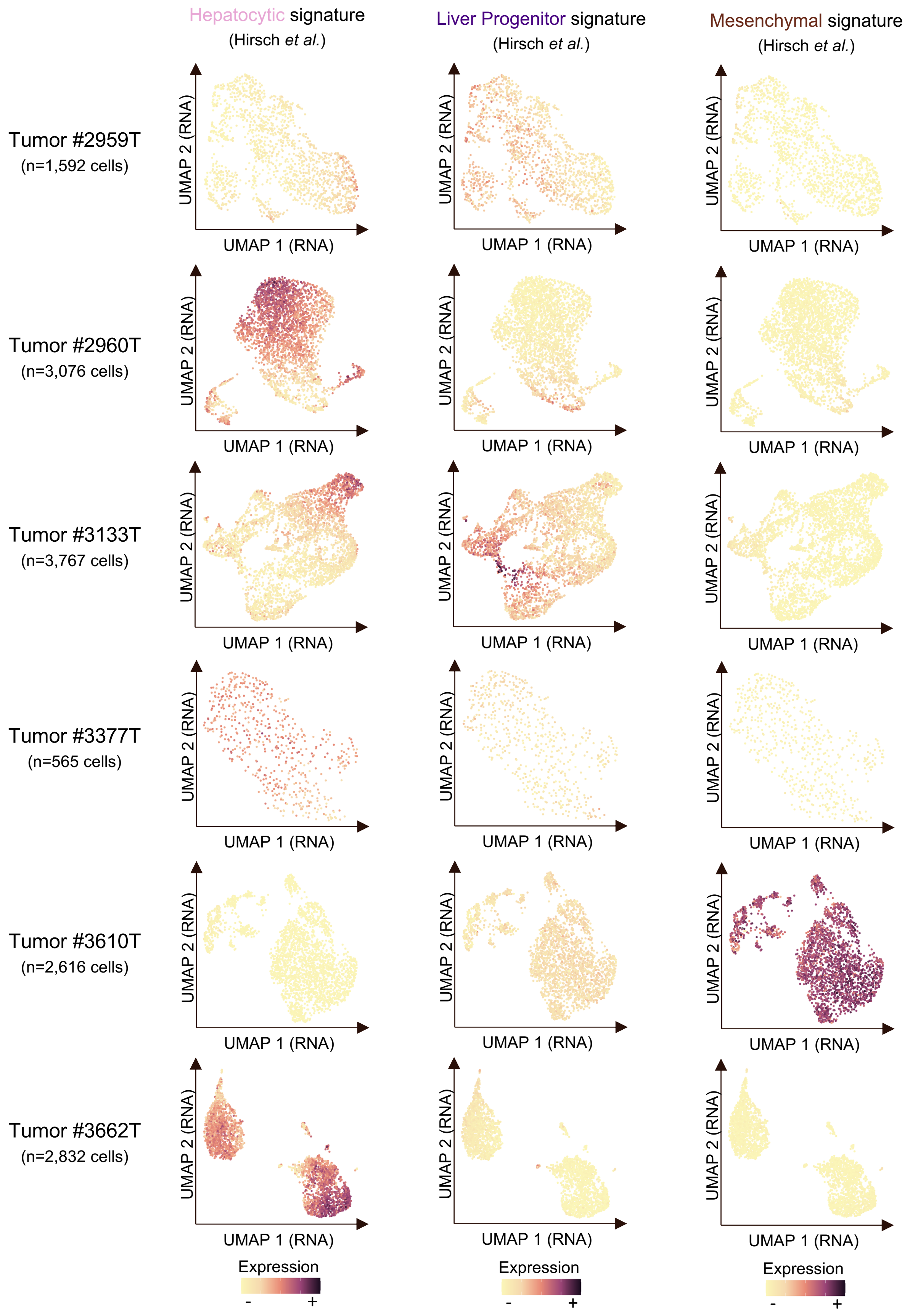

Supplementary Figure 6. Projection of HB subgroup signatures on individual tumor UMAPs. Each line shows the snRNA-seq UMAP of one tumor sample. The mean expression of marker genes of the Hepatocytic, Liver Progenitor and Mesenchymal subgoups, identified by bulk RNA-seq analysis of 100 HB (Hirsch *et al.*, 2021), are projected on each UMAP, from left to right.

Supplementary Figure 7

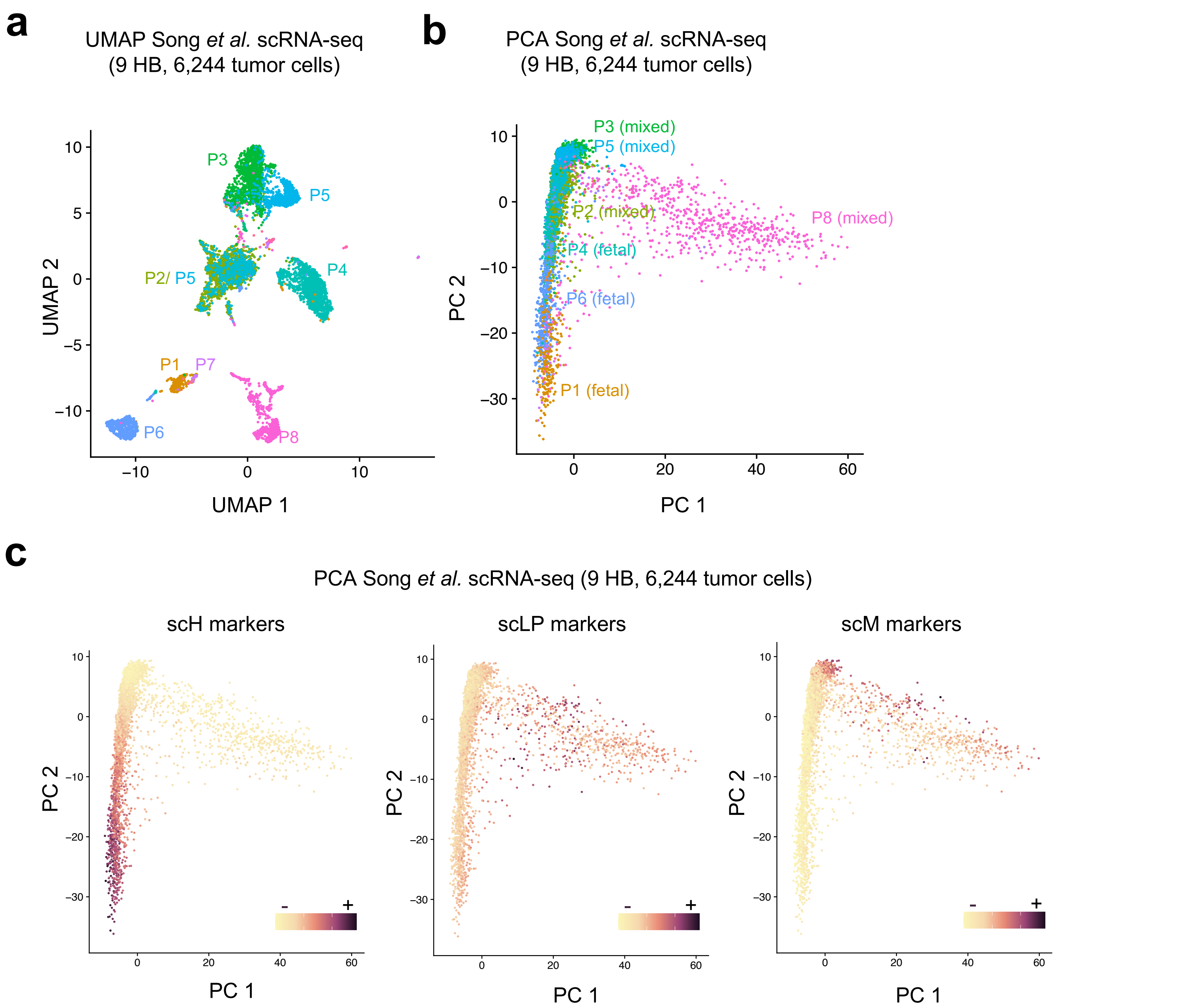

Supplementary Figure 7. UMAP and principal component analysis of Song's scRNA-seq data set. a UMAP visualization of 6,244 tumor cells from 9 HB show a grouping of cells by sample of origin. **b** Projection of HB cells on the first two principal components result in a mixture of cells from different samples. **c** Projection of HB cells on the first two principal components, with a color code showing the expression of scH, scLP and scM markers.

Supplementary Figure 8

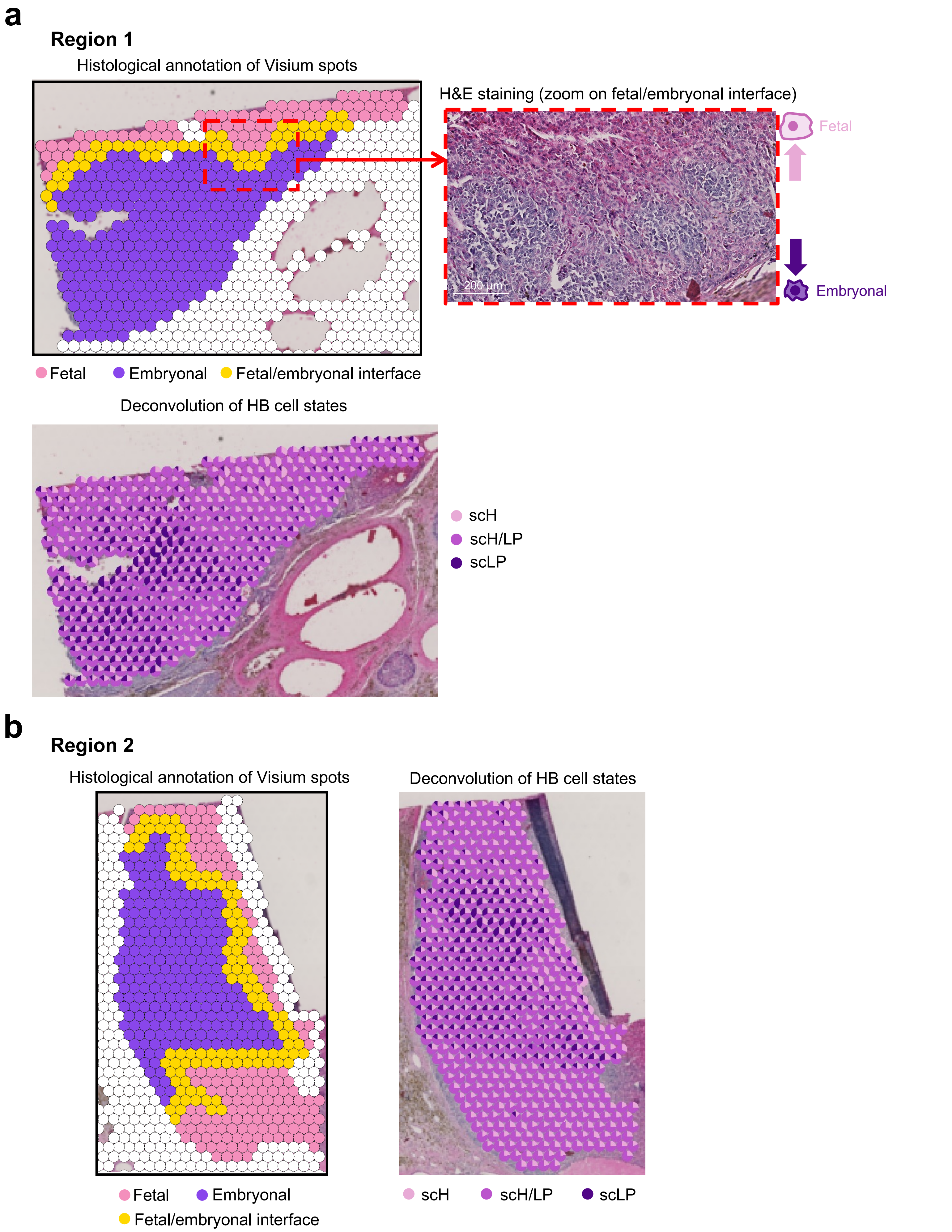

Supplementary Fig. 8. Deconvolution of HB cell states in spatial transcriptomics data. a Deconvolution of HB cell states in region 1. The upper left panel shows the histological annotations of spots. The upper right panel shows a zoomed-in focus on H&E staining of a region at the interface between fetal and embryonal cells. The bottom panel shows the results of the deconvolution, with a pie chart showing the estimated cell state proportions in each spot. **b** Deconvolution of HB cell states in region 2.

Supplementary Figure 9

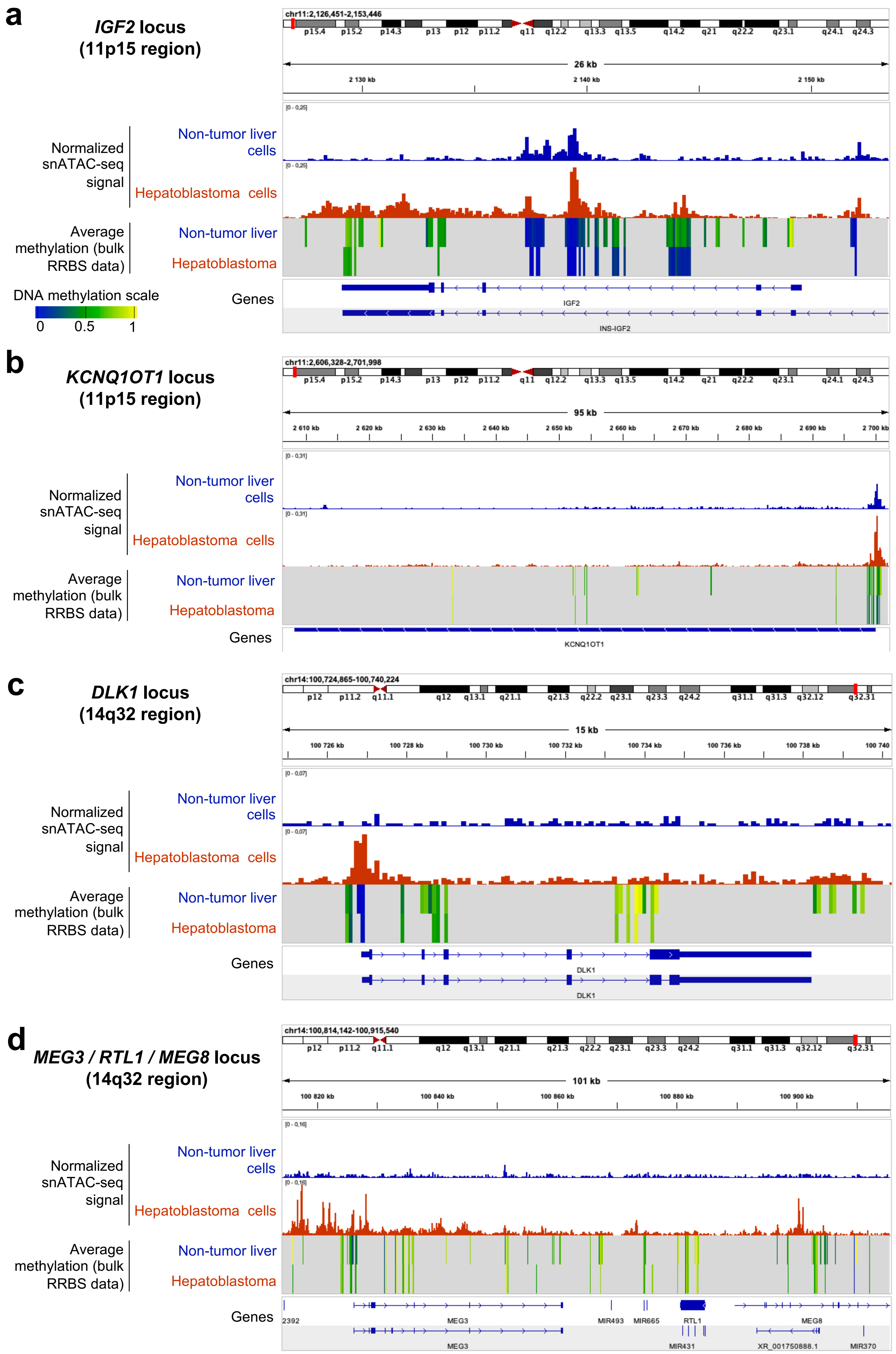

Supplementary Figure 9. Differential chromatin accessibility in imprinted regions. Shown are 4 loci (**a**, *IRF2*; **b**, *KCNQ1OT1*; **c**, *DLK1*; **d**, *MEG3/RTL1/MEG8*) located in imprinted regions implicated in HB development, with significant differentially accessible ATAC-seq peaks between HB and non-tumor (NT) liver cells. Upper tracks represent the normalized snATAC-seq signal in HB and NT cells. Lower tracks represent the methylation levels identified by bulk reduced representation bisulfite sequencing in Hirsch data set.

Supplementary Figure 10

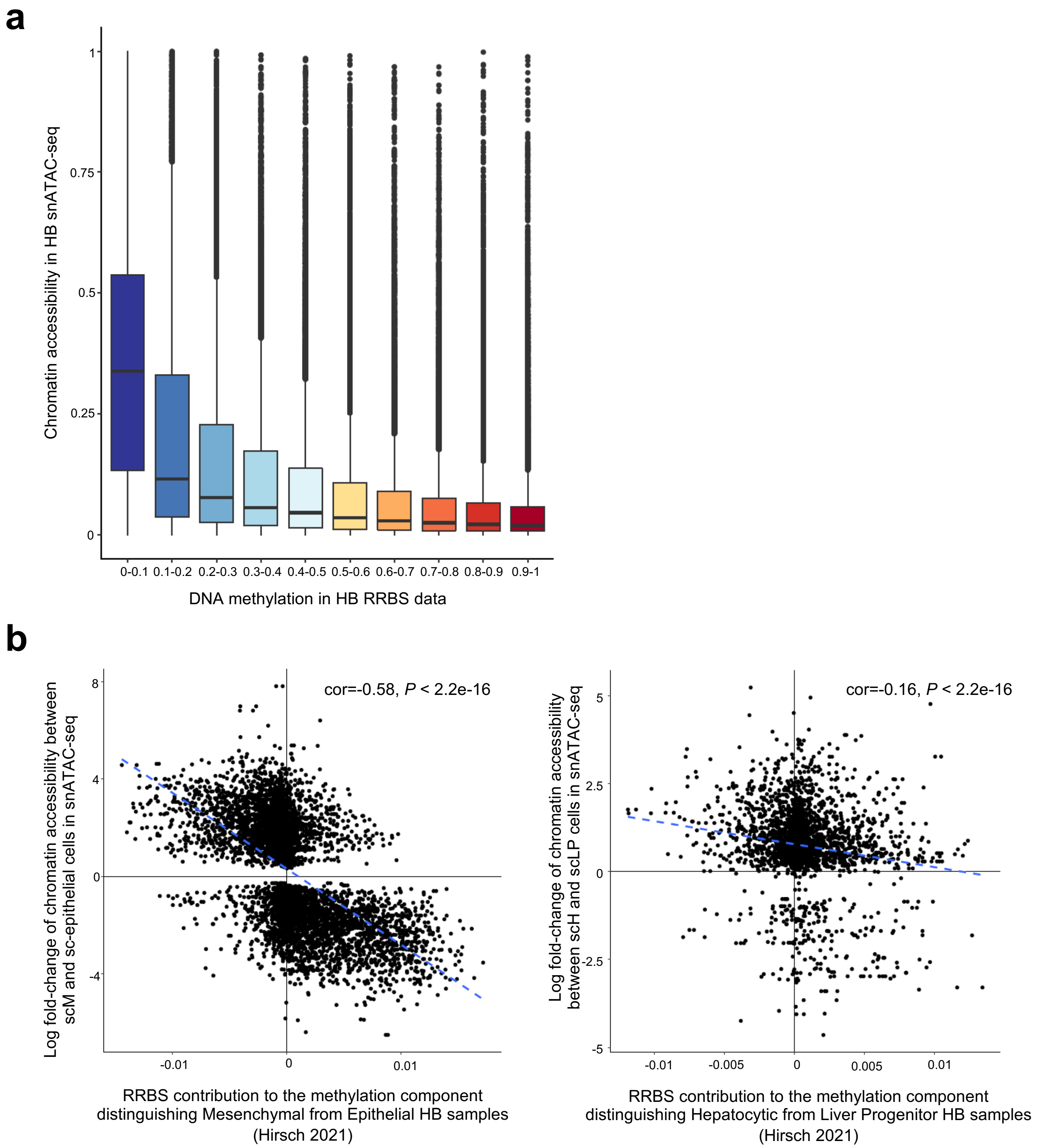

Supplementary Figure 10. Correlation between chromatin accessibility and DNA methylation. a Boxplot showing the distribution of chromatin accessibility in snATAC-seq as a function of the methylation of overlapping 100 bp tiles in reduced representation bisulfite sequencing (RRBS) data (Hirsch et al., Cancer Discov 2021). Middle bar, median; box, interquartile range; bars extend to 1.5 times the interquartile range. **b** Correlation (Pearson's correlation test) between the contribution of 100 bp tiles to DNA methylation components distinguishing HB subtypes and the chromatin accessibility changes observed between the same subtypes. Left: Mesenchymal vs. epithelial. Right: Hepatocytic vs. Liver Progenitor. Source data are provided in the Source Data file.

Supplementary Figure 11

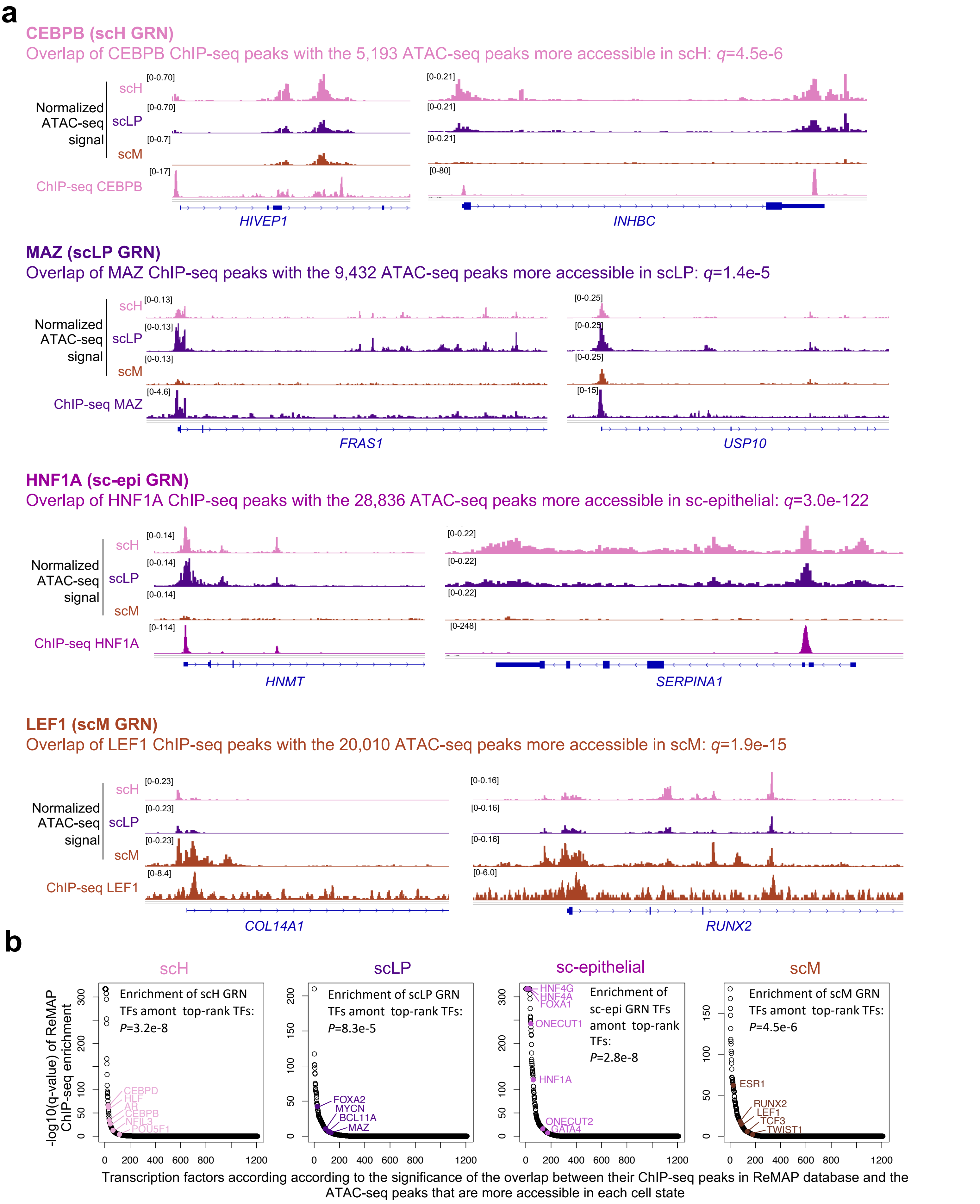

Supplementary Figure 11. Overlap between differentially accessible ATAC-seq peaks and transcription factor ChIP-seq peaks. a Eight representative regions are shown, displaying the overlap between ChIP-seq peaks of 4 key transcriptions factors (HNF1A associated with sc-epithelial cells, LEF1 with scM cells, CEBPB with scH cells and MAZ with scLP cells), together with the normalized ATAC-seq signals in scH, scLP and scM cells. All significant associations are reported in Sup Table 7. ChIP-seq coverage tracks were obtained from ENCODE (HNF1A in HepG2 cells: ENCFF502ACF; CEBPB in HepG2 cells: ENCFF406BBU; MAZ in HepG2 cells: ENCFF527EZL) or GEO (LEF1 in hESC: GSM1579343). **b** Distribution of GRN TFs among ReMAP ChIP-seq peaks enriched in the differential ATAC-seq peaks of each cell state. P-values were obtained using Wilcoxon's rank sum test (two-sided).

Supplementary Figure 12

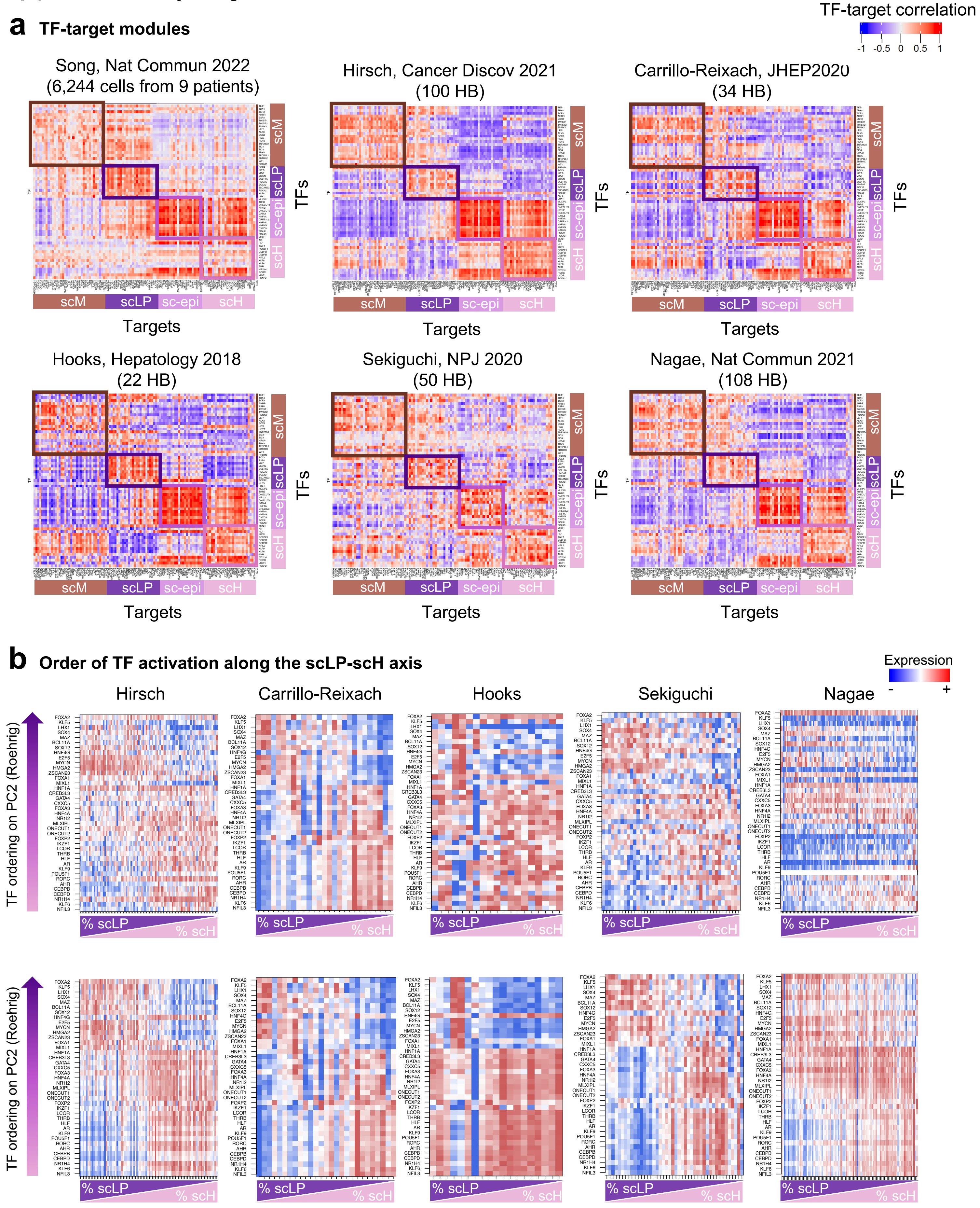

Supplementary Figure 12. Validation of GRNs in external data sets. a The correlation of the GRN transcription factors (TFs) and target genes were validated in one single-cell RNA-seq data set and 5 bulk RNA-seq data sets totalling 314 HB samples. Each heatmap shows the correlation between TFs (lines) and targets (columns), ordered as in Fig. 5a, in one of the series. **b** Activation of epithelial TFs in as a fonction of the proportion of scLP and scH cells estimated using Bisque deconvolution tool in 5 bulk RNA-seq series. TFs are ordered as in Fig. 6. Samples with a dominant scM population were excluded.

Supplementary Figure 13

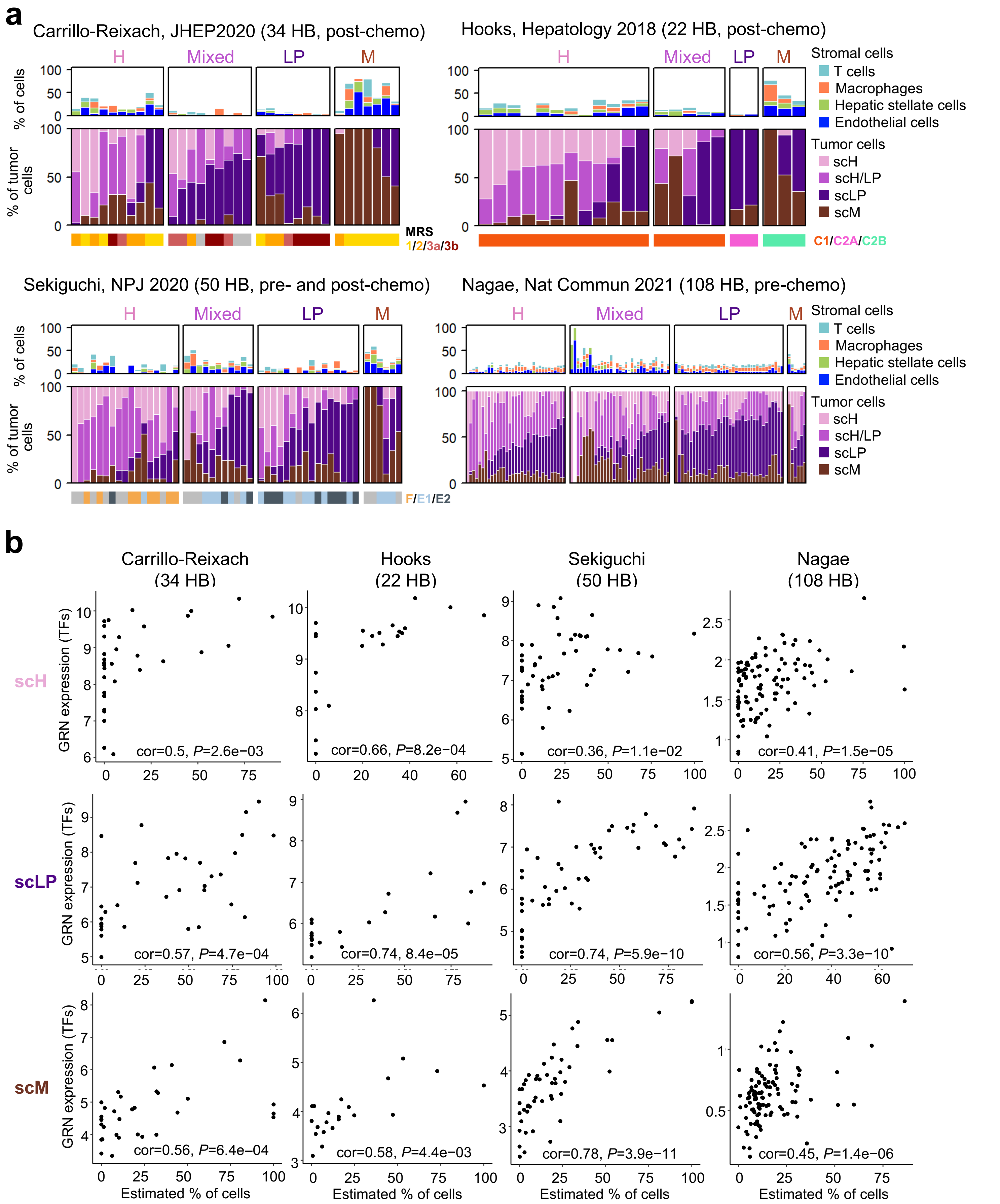

Supplementary Figure 13. Deconvolution of hepatoblastoma cell states in external data sets. a Deconvolution of HB cell states from bulk RNA-seq profiles of 214 HBs from 4 published series. Two barplots indicate the % of stromal cells (top) and tumor cell states (middle) in each sample. Samples are ordered according to their bulk transcriptomic subgroups (Hirsch's classification H/LP/M, assigned based on a hierarchical clustering of each cohort with the top 100 markers of each group). **b** Correlation (Pearson's correlation test) between the proportions of scH, scLP and scM cells estimated by Bisque and the expression of their GRNs in each data set. Source data are provided in the Source Data file.

Supplementary Figure 14

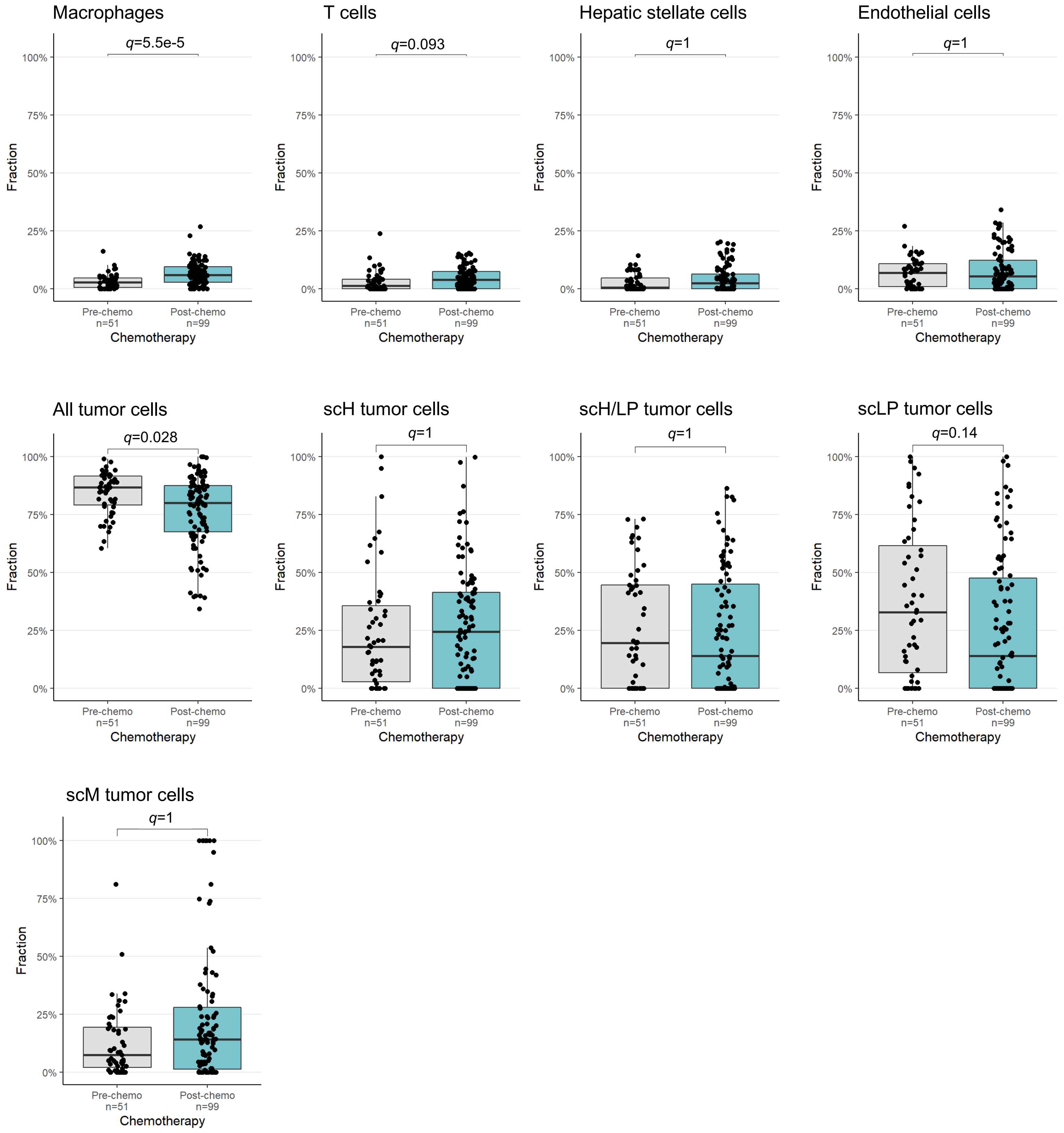

Supplementary Figure 14. Comparison of deconvoluted cell proportions between pre- and post-chemotherapy samples. We used bulk RNA-seq from two published series comprising both pre- and post-chemotherapy samples (Sekiguchi, NPJ 2020 & Hirsch, Cancer Discov 2021) to compare the abundance of stromal and tumor cells. Stromal and HB cell states were deconvoluted using Bisque tool. The proportions of each cell type were compared between 51 pre- and 99 post-chemotherapy samples using Wilcoxon's rank sum test (two-sided), with Bonferonni correction for multiple tesing. Source data are provided in the Source Data file. Middle bar, median; box, interquartile range; bars extend to 1.5 times the interquartile range.

Supplementary Figure 15

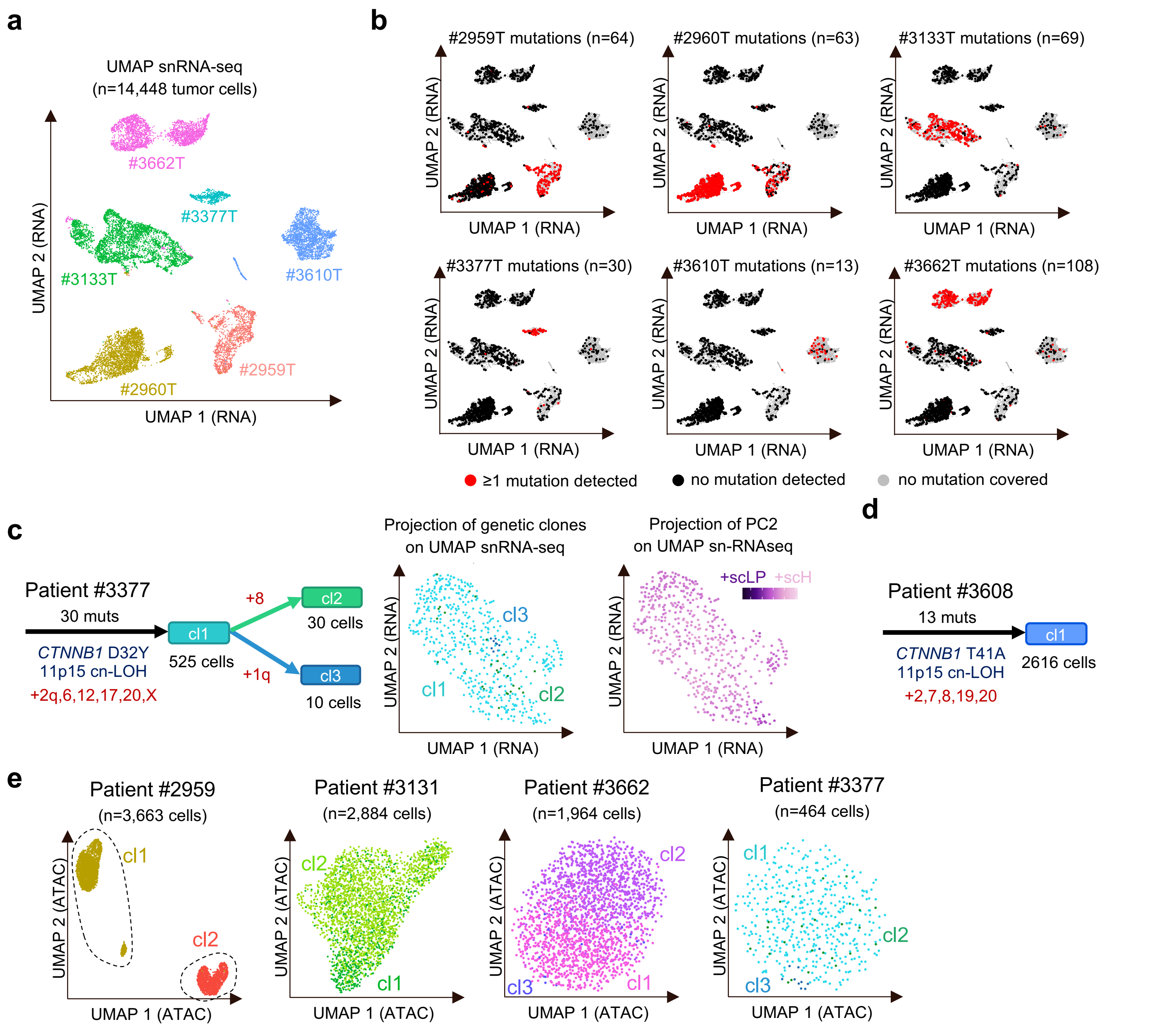

Supplementary Figure 15. Mapping genetic subclones in single-cell data. a Single-nucleus RNA-seq UMAP of the 14,448 tumor cells, colored by sample **b** Single-nucleus RNA-seq UMAPs of all tumor cells (as in **a**) with a color code indicating the somatic mutations detected in each cell. Each graph relates to the detection of somatic mutations from one sample. The number of WGS mutations detected in single-cell data is indicated in the title. Logically, part of #2959T mutations are found in #2960T (and vice versa), corresponding to common trunk mutations. **c** Tumor progression tree reconstructed for patient #3377 (left), with projection of genetic subclones (middle) and of PC2 contribution (right) on the snRNA-seq UMAP of tumor cells. **d** Tumor progression tree of tumor #3610T (patient #3608), in which no genetic subclone was identified. **e** Projection of genetic subclones on the snATAC-seq UMAP of each tumor.

Supplementary Figure 16

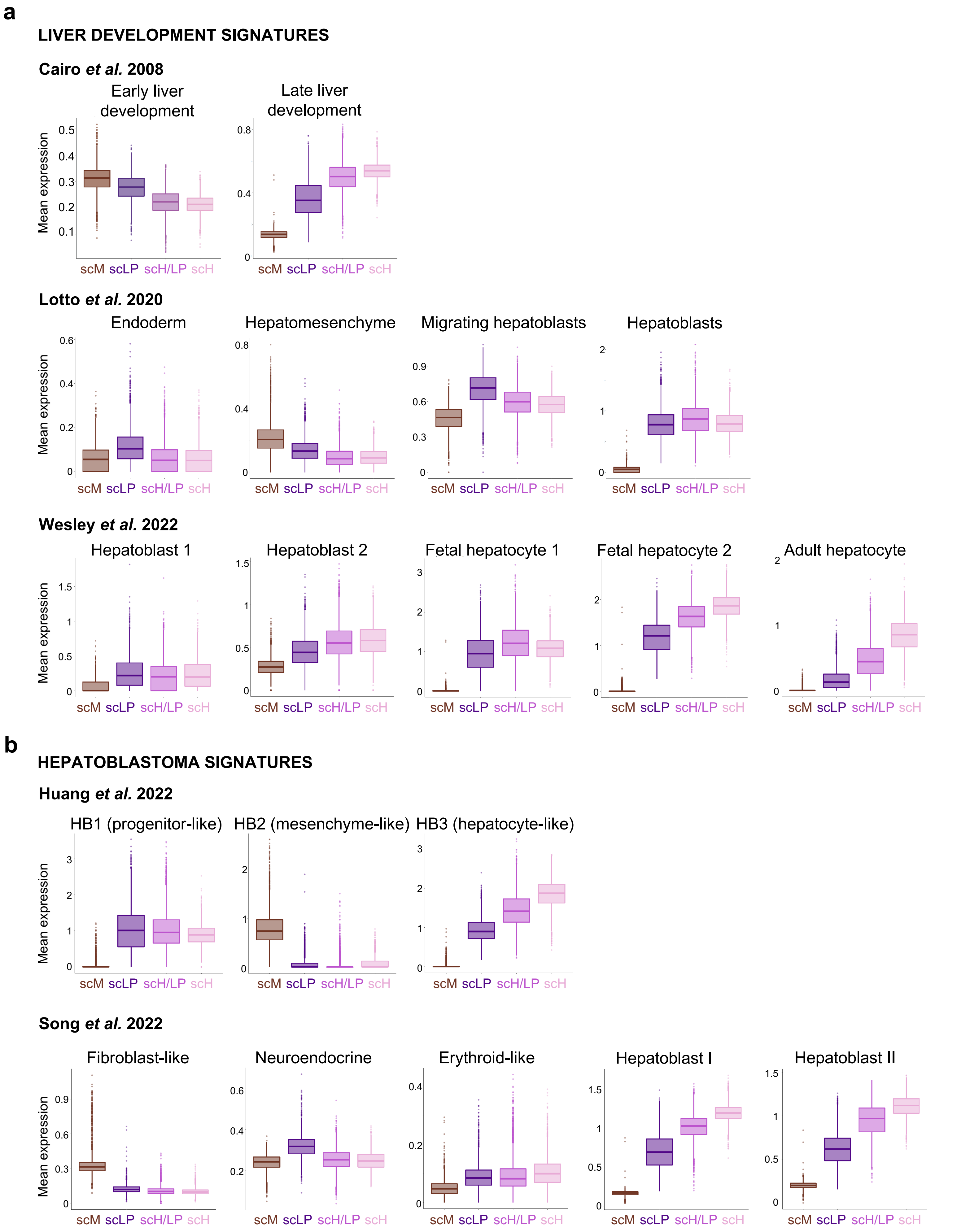

Supplementary Figure 16. Expression of liver development and hepatoblastoma signatures in single-cell states. a Box-and-whisker plots showing the distribution of the mean expression of published liver development signatures. Middle bar, median; box, interquartile range; bars extend to 1.5 times the interquartile range. **b** Same for published hepatolastoma signatures. Source data are provided in the Source Data file.
