# Supplementary material for: Single-cell multiomics reveals the interplay of clonal evolution and cellular plasticity in hepatoblastoma
Source: Nat Commun. 2024 Apr 8;15:3031. doi: 10.1038/s41467-024-47280-x (PMC11001886; doi:10.1038/s41467-024-47280-x)
Supplement: Supplementary file 3 — Description of Additional Supplementary Files [file 41467_2024_47280_MOESM3_ESM.pdf]

<u>Description of Additional Supplementary files</u>

Supplementary Data 1: Clinical, histological and molecular annotations of the Multiome cohort

Supplementary Data 2: Description of quality control thresholds and metrics for snRNA-seq and snATAC-seq

Supplementary Data 3: Top 120 markers for scH, scLP and scM cell subtypes and their detection in Visium

Supplementary Data 4: Number of differential ATAC-seq peaks for each comparison

Supplementary Data 5: Molecular description of the 158,707 chromatin accessibility peaks

Supplementary Data 6: ATAC-seq peaks in hepatoblastoma-related imprinted regions (11p15 and 14q32) showing significant accessibility changes in hepatoblastoma vs. non-tumor liver cells.

Supplementary Data 7: Expression of Gene Regulatory Networks (GRNs) transcription factors and target genes in differentiation poles

Supplementary Data 8: snRNA-seq expression correlations between the TFs and target genes of GRN modules
