# Supplementary material for: Single-cell multiomics reveals the interplay of clonal evolution and cellular plasticity in hepatoblastoma
Source: Nat Commun. 2024 Apr 8;15:3031. doi: 10.1038/s41467-024-47280-x (PMC11001886; doi:10.1038/s41467-024-47280-x)
Supplement: Supplementary file 12 — Reporting Summary [file 41467_2024_47280_MOESM12_ESM.pdf]

nature portfolio

Corresponding author(s): Jessica Zucman-Rossi & Eric Letouzé

Last updated by author(s): Mar 8, 2024

Reporting Summary

Nature Portfolio wishes to improve the reproducibility of the work that we publish. This form provides structure for consistency and transparency in reporting. For further information on Nature Portfolio policies, see our Editorial Policies and the Editorial Policy Checklist.

Statistics

For all statistical analyses, confirm that the following items are present in the figure legend, table legend, main text, or Methods section.

n/a	Confirmed	
☐	☒	The exact sample size (*n*) for each experimental group/condition, given as a discrete number and unit of measurement
☐	☒	A statement on whether measurements were taken from distinct samples or whether the same sample was measured repeatedly
☐	☒	The statistical test(s) used AND whether they are one- or two-sided<br>*Only common tests should be described solely by name; describe more complex techniques in the Methods section.*
☒	☐	A description of all covariates tested
☒	☐	A description of any assumptions or corrections, such as tests of normality and adjustment for multiple comparisons
☐	☒	A full description of the statistical parameters including central tendency (e.g. means) or other basic estimates (e.g. regression coefficient) AND variation (e.g. standard deviation) or associated estimates of uncertainty (e.g. confidence intervals)
☐	☒	For null hypothesis testing, the test statistic (e.g. *F*, *t*, *r*) with confidence intervals, effect sizes, degrees of freedom and *P* value noted<br>*Give P values as exact values whenever suitable.*
☒	☐	For Bayesian analysis, information on the choice of priors and Markov chain Monte Carlo settings
☒	☐	For hierarchical and complex designs, identification of the appropriate level for tests and full reporting of outcomes
☐	☒	Estimates of effect sizes (e.g. Cohen's *d*, Pearson's *r*), indicating how they were calculated

Our web collection on statistics for biologists contains articles on many of the points above.

Software and code

Policy information about availability of computer code

Data collection	We used 10X Genomics cellranger-arc (v2.0.0) software for raw data analysis.
Data analysis	As detailed in the Methods section, the following tools were used for data analysis:<br>Seurat (v3)<br>ArchR (v1.0.1)<br>Signac (v1.6)<br>InferCNV (v1.6)<br>spaceranger (v1.3.1)<br>screadcounts (v1.1.8)<br>samtools view (v1.14)<br>scPower (v1.0.4)<br>ReMapEnrich (v0.99.0)<br>deconverse (v0.2.0)<br>SPOTlight (v1.5.1)<br><br>Custom R scripts generated for this study are available on Github: https://github.com/FunGeST/Roehrig2023_HB_plasticity_scripts

For manuscripts utilizing custom algorithms or software that are central to the research but not yet described in published literature, software must be made available to editors and reviewers. We strongly encourage code deposition in a community repository (e.g. GitHub). See the Nature Portfolio guidelines for submitting code & software for further information.

Data

Policy information about availability of data

All manuscripts must include a data availability statement. This statement should provide the following information, where applicable:

- Accession codes, unique identifiers, or web links for publicly available datasets
- A description of any restrictions on data availability
- For clinical datasets or third party data, please ensure that the statement adheres to our policy

Raw sequencing data from the single-cell Multiome and spatial transcriptomics experiments performed for this study have been deposited to the European Genome Archive (EGA) under accession code EGAS00001006932 [https://web2.ega-archive.org/studies/EGAS00001006932]. These data contain identifiable genetic variants and are thus accessible under controlled access for patient privacy concerns, by contacting the data access committee. We also re-analyzed single-cell RNA-seq data from Song et al. (GEO database accession [https://www.ncbi.nlm.nih.gov/geo/query/acc.cgi?acc=GSE186975]) and bulk RNA-seq from Hirsch et al. (EGA accession [https://web2.ega-archive.org/studies/EGAS00001005108]), Nagae et al., (NBDC Human Database accession [https://humandbs.biosciencedbc.jp/en/hum0233-v1]), Hooks et al. (GEO database accession [https://www.ncbi.nlm.nih.gov/geo/query/acc.cgi?acc=GSE104766]), Sekiguchi et al. (Japanese Genotype-phenotype Archive accession [https://humandbs.biosciencedbc.jp/en/hum0035-v5#JGAS000088R]) and Carrillo-Reixach et al. (GEO accession [https://www.ncbi.nlm.nih.gov/geo/query/acc.cgi?acc=GSE132219]). ChIP-seq bigwig files were downloaded from ENCODE (https://www.encodeproject.org/files/ENCFF502ACF/; https://www.encodeproject.org/files/ENCFF406BBU/; https://www.encodeproject.org/files/ENCFF527EZL/) or GEO (https://www.ncbi.nlm.nih.gov/geo/query/acc.cgi?acc=GSM1579343). Source data of Figures and Supplementary Figures have been provided as Source Data files.

Research involving human participants, their data, or biological material

Policy information about studies with human participants or human data. See also policy information about sex, gender (identity/presentation), and sexual orientation and race, ethnicity and racism.

Reporting on sex and gender	The biological sex of each patient is reported in Supplementary Table 1.
Reporting on race, ethnicity, or other socially relevant groupings	Not applicable.
Population characteristics	Age at diagnosis, surgery and outcome are reported in Supplementary Table 1.
Recruitment	We selected 6 hepatoblastoma samples representative of hepatoblastoma diversity based on their bulk RNA-seq profiles and histological block reviewing. This series includes samples of the Hepatocytic (n=2), Liver Progenitor (n=3) and Mesenchymal (n=1) transcriptomic subgroups. Two synchronous samples correspond to LP (#2959T) and H (#2960T) regions of the same tumor. A limitation of this study is the small sample size. Single-cell multiomic analyses of larger series may reveal additional HB cell states not represented in the current data set.
Ethics oversight	The study was approved by the local Ethics Committee (CCPRB Paris Saint-Louis).

Note that full information on the approval of the study protocol must also be provided in the manuscript.

Field-specific reporting

Please select the one below that is the best fit for your research. If you are not sure, read the appropriate sections before making your selection.

☒ Life sciences ☐ Behavioural & social sciences ☐ Ecological, evolutionary & environmental sciences

For a reference copy of the document with all sections, see nature.com/documents/nr-reporting-summary-flat.pdf

Life sciences study design

All studies must disclose on these points even when the disclosure is negative.

Sample size	We conducted in-depth analysis of 6 hepatoblastomas by whole genome sequencing and single-nucleus Multiome. This sample size allowed us to include samples representative of the 3 main transcriptomic subgroups. The findings were validated in external data (9 single-cell RNA-seq and 314 bulk RNA-seq samples), justifying that the sample size was sufficient to identify robust gene regulatory networks of the main hepatoblastoma subtypes. Future studies of larger series may reveal rare subtypes not covered in the present data.
Data exclusions	We removed poor quality cells as explained in the Methods section.
Replication	Gene expression signatures were validated in a bulk RNA-seq series of 314 hepatoblastomas.
Randomization	Not applicable. This study does not compare two treatments but explores the diversity of hepatoblastoma subtypes.
Blinding	Blinding was not feasible given that the 6 samples were carefully selected based on previous molecular and histological data. However, all samples were analyzed using the same scripts, precluding bias in data processing.

Reporting for specific materials, systems and methods

We require information from authors about some types of materials, experimental systems and methods used in many studies. Here, indicate whether each material, system or method listed is relevant to your study. If you are not sure if a list item applies to your research, read the appropriate section before selecting a response.

Materials & experimental systems

n/a	Involved in the study
☒	☐ Antibodies
☒	☐ Eukaryotic cell lines
☒	☐ Palaeontology and archaeology
☒	☐ Animals and other organisms
☒	☐ Clinical data
☒	☐ Dual use research of concern
☒	☐ Plants

Methods

n/a	Involved in the study
☒	☐ ChIP-seq
☒	☐ Flow cytometry
☒	☐ MRI-based neuroimaging
